# Next-generation red ultra-bright fluorescent dyes for nuclear imaging and peripheral blood leukocytes sorting†

**DOI:** 10.1039/d4sc04848b

**Published:** 2024-09-27

**Authors:** Zipeng Li, Zheng Liu, Ding Yu, Qichao Yao, Wanying Ma, Changyu Zhang, Jiangli Fan, Xiaojun Peng

**Affiliations:** a State Key Laboratory of Fine Chemicals, Frontiers Science Center for Smart Materials Oriented Chemical Engineering, Dalian University of Technology Dalian 116024 China fanjl@dlut.edu.cn; b Ningbo Institute of Dalian University of Technology Ningbo 315016 China; c Liaoning Binhai Laboratory Dalian 116023 China

## Abstract

The nucleus is a membrane-bound organelle in eukaryotic cells and plays a crucial role in cellular processes. Visualizing nuclear morphology is essential for investigating nuclear functions and understanding the relationship between nuclear morphological alterations and multiple diseases. Fluorescent dyes have been developed to visualize nuclear morphology, but the selection of red nuclear-labeling fluorescent dyes remains limited (high price, unknown structure, or high toxicity). Herein, we have developed a red ultra-bright nuclear-targeted dye, BPC1, through the engineering of unsymmetrical cyanine dyes derived from D–π–A systems. BPC1 exhibits ultrahigh fluorescence brightness and exceptional cell permeability, and selectively stains nuclear DNA rather than mitochondrial DNA, enabling the visualization of the nucleus in diverse cells at extremely low doses (100 nM) and laser power (0.8 μW). Furthermore, BPC1 is utilized for nuclear staining in blood cells, aiding in the distinct visualization of the white blood cell nucleus and facilitating the identification and enumeration of various leukocyte types. Our study implies considerable commercial potential for BPC1 and underscores its capacity to serve as a powerful tool in life sciences and cell biology research.

## Abstract 2

The red ultra-bright nuclear-targeted dye BPC1 was developed. BPC1 exhibits ultrahigh fluorescence brightness and exceptional cell permeability, enabling the visualization of nucleus by substantially lower doses (100 nM) and lower laser power.

## Graphical abstract

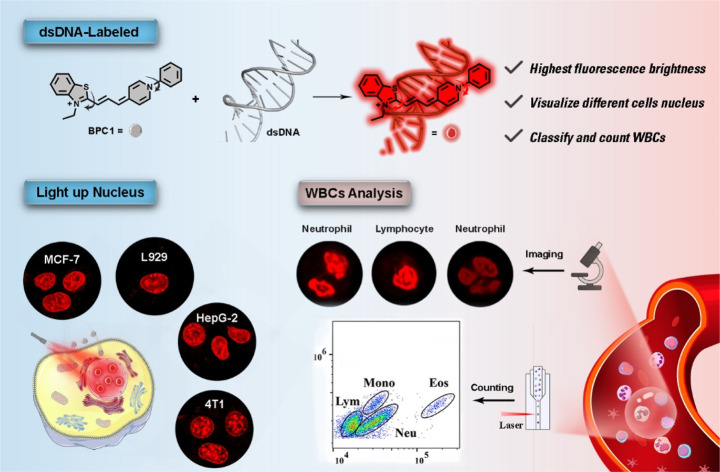

## 1 Introduction

The nucleus is a membrane-bound organelle in eukaryotic cells and controls key cellular processes including gene expression, DNA replication, and cell division. The notable characteristic of the nucleus is plasticity, as the diverse environment can influence its resting state (shape, size and contents).^1–3^ The nuclear morphology and chromatin are considered as markers of cellular physiological activity and associated with numerous human diseases such as cancer, neurodegenerative disorders, and aging.^4–7^ Fluorescence imaging technology possesses features of non-invasiveness, real-time feedback, high sensitivity, and superior spatial resolution, granting it indispensable advantages in nuclear detection.^8–12^ The most direct strategy involves using genetically encoded fluorescent proteins to label nuclear proteins.^13,14^ While this method offers high precision, it is limited by factors such as low transfection efficiency, protein overexpression, and complex procedures. Nuclear DNA fluorescent dyes with high sensitivity and signal-to-noise ratio have been developed.^15,16^ However, dyes intended for DNA staining commonly exhibit nonspecific binding with RNA, because the variance in base pairing between DNA and RNA is minimal, and both molecules exhibit major and minor grooves within their secondary structures.^17–20^ Moreover, a small quantity of mitochondrial DNA is present in the cytoplasm.^21–23^ The coexistence of DNA in the cell nucleus and cytoplasm poses a challenge in the development of targeted fluorescent dyes for nuclear DNA staining.

To date, DNA-specific blue fluorescent dyes such as DAPI and Hoechst are still widely favored for nuclear labeling due to their simplicity, cost-effectiveness, and independence from genetic modification requirements.^24,25^ However, they rely on high-energy ultraviolet laser excitation, potentially causing DNA damage and cell death.^26–28^ In recent years, there has been a substantial increase in the demand for long-wavelength fluorescent dyes, particularly in the Red/Near-Infrared Region (NIR).^29,30^ In contrast to short-wavelength fluorescent dyes, these dyes exhibit reduced spontaneous fluorescence interference and enable deeper tissue imaging. However, the selection of commercially available red nuclear-labeling fluorescent dyes remains limited. To date, SYTO Deep Red from Thermo Fisher Scientific exhibits high usage costs and an undisclosed structure. DRAQ5, due to its notorious high toxicity, is gradually being phased out.^31,32^ Recent studies have shown that conjugating visible light-excited fluorochromes to the Hoechst*via* linker groups is a frequently used strategy to construct red nuclear-labeling fluorescent dyes.^33,34^ Alternatively, the elongation of the conjugate chain length in thiazole orange dyes allows for the generation of red fluorescent dyes that exhibit selective binding to cell nuclear DNA.^35–38^ However, their affinity for binding to nuclear DNA is generally low, leading to nonspecific retention by mitochondria. Therefore, nuclear labeling dyes are urgently needed to match the increasing scope and precision of the studies in nuclear biology.

**Fig. 1:**
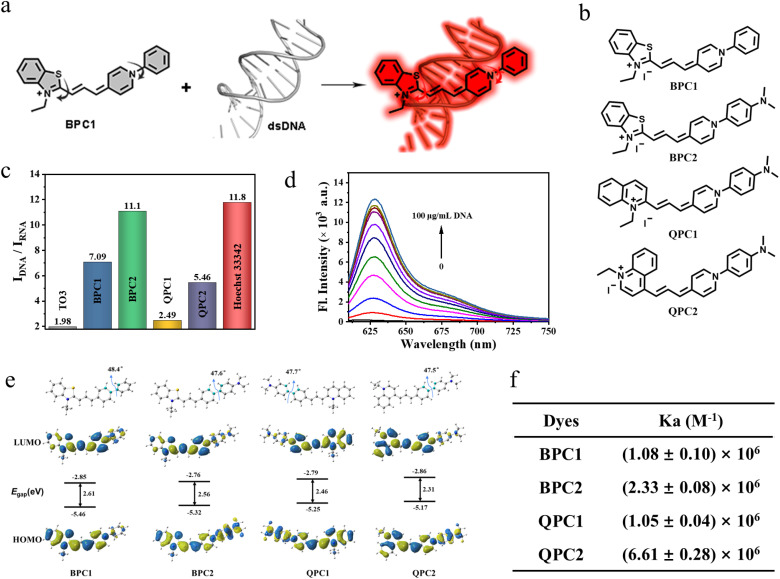
(a) Schematic illustration of nuclear targeted DNA marker dyes by combining dsDNA. (b) Structures of BPC1, BPC2, QPC1 and QPC2. (c) The ratio of maximal fluorescence intensities for different dyes with CT DNA or yeast RNA. (d) Fluorescence spectrum of concentration titration for BPC1 (4 μM) with different concentrations of CT DNA (0–100 μg mL^−1^). (e) Optimized geometry and the HOMO/LUMO of BPC1, BPC2, QPC1, and QPC2 in the ground state at the B3LYP/6-31G* level. (f) dsDNA binding efficiency of BPC1, BPC2, QPC1 and QPC2.

In this study, a series of unsymmetrical cyanine dyes with different donor–π–acceptor (D–π–A) systems were designed (BPC1, BPC2, QPC1 and QPC2, Fig. 1b). The absorption wavelengths of these dyes range between 550 nm and 700 nm, and their inherent fluorescence is minimal due to molecular rotation (Fig. 1a). Once upon binding to DNA, their fluorescence is significantly enhanced to varied degrees. Among these dyes, the benzothiazole *N*-aryl pyridinium cyanine dye (BPC1) specifically bound to DNA and exhibited red emission without being influenced by other biomolecules. It displays ultrahigh fluorescence brightness and exceptional cell permeability and selectively stains nuclear DNA rather than mitochondrial DNA, enabling real-time visualization of the nucleus by 3D imaging, super-resolution fluorescence imaging, and fluorescence lifetime imaging at extremely low doses (100 nM) and laser power (0.8 μW). Due to the excellent nuclear targeting ability, the use of BPC1 for nuclear staining of blood cells can effectively distinguish various leukocyte nuclei and facilitate the classification and counting of different types of leukocytes.

## 2 Results and discussion

### 2.1 Design and synthesis

Thiazole-Orange-3 (TO3) dye exhibits favorable properties for nucleic acid response. However, its insufficient DNA specificity (Fig. S1b and e†) and inadequate nuclear pores permeability restrict its use as DNA labeling dye for nuclear labeling (Fig. S8†). Thus, we replaced the D–π–A scaffold of TO3 with different nitrogen-containing heterocycles (benzothiazole, *N*-arylpyridine, and quinoline), and then generated BPC1, BPC2, QPC1 and QPC2 with an appropriate conjugated length and a molecular skeleton that can be twisted (Fig. 1b). We envision that these modifications can change the DNA specificity and nuclear pore permeability.^39^ These candidate dyes were synthesized *via* similar processes. First, the quaternary ammonium salt condenses with *N*,*N*-diphenylformamidine to obtain an asymmetric cyanine dye intermediate, and then further condensed with another part of the quaternary ammonium salt to obtain the corresponding asymmetric cyanine dyes BPC1, BPC2, QPC1 and QPC2. The specific synthesis process and chemical structure characterization (^1^H NMR, ^13^C NMR, and HR-MS) are shown in Schemes S1–S7 and Fig. S15–S26 (ESI).†

### 2.2 Photophysical properties

The photophysical properties of BPC1, BPC2, QPC1 and QPC2 were first investigated in phosphate-buffered solution (PBS) at pH 7.4. As shown in Fig. S1a–l and Table S1,†BPC1, BPC2, QPC1 and QPC2 exhibited maximal absorption at 580 nm, 580 nm, 615 nm and 665 nm with negligible fluorescence emission, respectively. By contrast, after addition of calf thymus DNA (CT DNA), they showed red-shifted absorption peaks (BPC1: 600 nm, BPC2: 605 nm, QPC1: 635 nm, QPC2: 690 nm) alongside a notable fluorescence emission peak (BPC1: 627 nm, BPC2: 637 nm, QPC1: 665 nm, QPC2: 715 nm), while the addition of yeast RNA resulted in only a marginal enhancement of the fluorescence signal. The fluorescence quantum yield and lifetime of dyes were tested in the absence or presence of nucleic acid (CT DNA or yeast RNA). It was observed that the addition of CT DNA notably increased the quantum yield and prolonged the fluorescence lifetime to varied extents (Tables S1, S2 and Fig. S2†). In contrast, the extension in the fluorescence lifetime occurred in the presence of yeast RNA, yet the increase in quantum yield was minimal. By calculating the alterations in radiative and non-radiative rate constants of each dye pre- and post-binding to CT DNA or yeast RNA (Table S2†), it was determined that the radiative rate constants of dyes significantly increased after binding to CT DNA (BPC1: 1.55 × 10^8^ s^−1^, BPC2: 1.84 × 10^8^ s^−1^, QPC1: 6.71 × 10^7^ s^−1^, QPC2: 7.29 × 10^7^ s^−1^), surpassing those after yeast RNA binding (BPC1: 4.00 × 10^7^ s^−1^, BPC2: 4.23 × 10^7^ s^−1^, QPC1: 2.24 × 10^7^ s^−1^, QPC2: 2.59 × 10^7^ s^−1^). Therefore, the fluorescence quantum yield was much higher in the presence of CT DNA than yeast RNA. In addition, the fluorescence brightness of the dyes bound to CT DNA was calculated using a specific formula (*ε*_max_ × *Φ*_F_). BPC1 exhibited the highest fluorescence brightness (BPC1: 71 820, BPC2: 40 752, QPC1: 2408, QPC2: 9216). To our knowledge, it's the brightest DNA-binding dye discovered thus far (Table S2†).

We further investigated the discriminatory capacity of the dyes for DNA/RNA by comparing the fluorescence intensity after binding with DNA or RNA. As shown in Fig. 1c and Table S1,† the I_DNA_/I_RNA_ of BPC1, BPC2, QPC1, and QPC2 for nucleic acids was 7.09, 11.10, 2.49, 5.46, respectively, which was significantly higher than that of TO3 (1.98) and SYTO Deep Red (3.54, commercial red DNA-labeling dye). Additionally, these four dyes show negligible fluorescence over time. In the presence of DNA, the fluorescence intensities reached their maximum plateau within seconds and remained stable for the next 5 minutes, indicating their rapid binding with DNA (Fig. S4†). And there is a good correlation between the fluorescence intensity and DNA concentrations (0–100 μg mL^−1^) (Fig. 1d and S5†). The affinity of these four dyes to DNA was (1.08 ± 0.10) × 10^6^ M^−1^, (2.33 ± 0.08) × 10^6^ M^−1^, (1.05 ± 0.04) × 10^6^ M^−1^ and (6.61 ± 0.28) × 10^6^ M^−1^, respectively (Fig. 1f and S3†), indicating that they all have a high affinity for DNA. These results indicate that the synthesized series of pyridine cyanine dyes have the potential for quantitative detection of DNA concentration.

### 2.3 Gaussian calculation

Considering the notable disparities in the optical and chemical characteristics of the pyridine cyanine dyes, we conducted Density Functional Theory (DFT) and Time-Dependent Density Functional Theory (TD-DFT) calculations using the B3LYP function and the 6-31G* basis set. Fig. 1e and Table S4† depict the Highest Occupied Molecular Orbitals and Lowest Unoccupied Molecular Orbitals (HOMOs and LUMOs). All four dyes exhibited a similar dihedral angle of approximately 48° between the phenyl moiety and the pyridine moiety, as observed in the optimized geometry. The larger dihedral angle caused the molecule to undergo non-radiative transitions back to the ground state from the excited state, resulting in fluorescence quenching. Additionally, the energy gap between the HOMO and LUMO decreased with the red shift in absorption of the dyes BPC1, BPC2, QPC1, and QPC2, consistent with the experimental test results.

### 2.4 Selectivity and binding constant

**Fig. 2:**
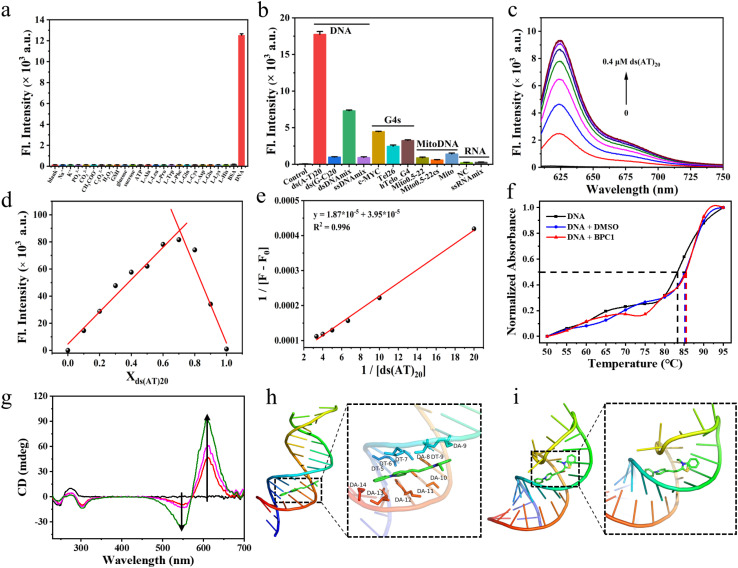
(a) Fluorescence intensity responses of BPC1 (4 μM) at 625 nm to varying substances. (b) Fluorescence intensity responses of BPC1 (1 μM) at 625 nm to varying oligonucleotides (4 μM). (c) Fluorescence spectral responses of BPC1 to varying concentrations of ds(AT)_20_ DNA. (d) Job's plot for BPC1 and ds(AT)_20_ DNA. *X*-axis represents the mole fraction of ds(AT)_20_ DNA. The total concentration for BPC1 and ds(AT)_20_ DNA was fixed to 10 μM. (e) Benesi–Hildebrand plots of the reciprocal changes in fluorescence intensity at 625 nm as a function of the reciprocal ds(AT)_20_ DNA. (f) Melting temperature (*T*_m_) profiles of CT DNA (10 μg mL^−1^) under different conditions: blank, BPC1 (4 μM, 4 μL), and DMSO (4 μL). (g) CD spectra of CT DNA (0.5 mg mL^−1^) to varying concentrations of BPC1 (0–0.2 mM). Molecular docking calculation of BPC1 with (h) dsDNA (3FDQ) or (i) ssRNA (6UGI).

Given its superior performance in terms of fluorescence quantum yield (54%), signal-to-noise ratio (7.09), and brightness (71 820) following DNA binding, we chose BPC1 as the primary focus of our subsequent research. Considering that the intracellular microenvironment is complex and contains many active substances, the ability of BPC1 to resist interference is an important indicator for its biological applicability. Therefore, the specificity of BPC1 for DNA was investigated against various analytes including ions (Na^+^, K^+^, PO_4_^3−^, CO_3_^2−^, CH_3_COO^−^, and C_2_O_4_^2−^), reactive species (H_2_O_2_ and GSH), sugars (glucose and sucrose), ATP, various amino acids (l-Ala, l-Leu, l-Pro, l-Trp, l-Phe, l-Gln, l-Cys, l-Asp, l-Glu, and l-His), and Bovine Serum Albumin (BSA). As shown in Fig. 2a, the addition of each analyte at a concentration of 100 μM resulted in negligible interference. Specifically, BSA tends to trigger false positive signals with its hydrophobic pockets,^40^ yet BPC1 exhibits little non-specific binding for BSA. These findings demonstrated the high specificity of BPC1 for DNA, making it well-suited for studying nucleic acids within cells without interference from other biomolecules.

Furthermore, we studied the binding capability of BPC1 to different types of nucleic acids (Table S5† and Fig. 2b). We found that the fluorescence signal of BPC1 almost did not increase upon addition of RNA (NC and ssRNAmix). Upon addition of several atypical DNAs (mitochondrial DNA, G4-DNA, and ssDNA), a weak fluorescence enhancement was observed. In contrast, significant fluorescence enhancements were observed upon the addition of dsDNAmix, and the fluorescence intensity increased more notably following the addition of specific DNA sequences ds(AT)_20_ than ds(GC)_20_. And the fluorescence intensity of BPC1 was linearly correlated with the concentration of typical dsDNA (ds(AT)_20_ and ds(GC)_20_) (Fig. 2c and S6a†). Furthermore, the Job plot analysis showed that BPC1 interacted with ds(AT)_20_ in a 1 : 2 stoichiometric ratio, and the binding constant was estimated to be (2.11 ± 0.54) × 10^−6^ M^−1^ (Fig. 2d, e, S6b and c†). BPC1 interacted with ds(GC)_20_ in a 1 : 1 stoichiometric ratio, and the binding constant is (7.27 ± 1.87) × 10^−5^ M^−1^. Thus Ka(AT)_20_ was approximately an order of magnitude higher than Ka(GC)_20_. All the results indicate that BPC1 tends to bind to AT base pairs.

### 2.5 Binding mechanism

To determine the binding mode of BPC1 with nucleic acids, thermal melting analysis was conducted (Fig. 2f). The results indicated that DMSO had no significant effect on the Tm of dsDNA, and the addition of 4 μM BPC1 did not alter the *T*_m_ either, suggesting a non-intercalative binding mode of BPC1 with dsDNA. The concentration-dependent CD spectra of BPC1 (0–0.2 mM) in a fixed concentration of dsDNA (0.5 mg mL^−1^) revealed positive signals at 280 nm, negative signals at 245 nm, and an Induced Circular Dichroism (ICD) signal enhancement at 550 nm and 610 nm, indicating a groove binding mode of BPC1 with dsDNA (Fig. 2g). Molecular docking simulations were further used to reveal the mechanism of BPC1 with nucleic acids. X-ray crystal structures of dsDNA (3FDQ) and ssRNA (6UGI) were obtained from the PDB database. Subsequently, docking with BPC1 was performed using the Local Search Parameters module of AutoDock 4.2. The results showed that BPC1 had a higher binding energy (8.5 kcal mol^−1^) with dsDNA than with dsRNA (8.0 kcal mol^−1^) (Table S6†). Taking a closer look at the detailed binding modes (Fig. 2h), BPC1 adopted a planar conformation to bind to the minor groove of dsDNA, surrounded by the nucleotides DT (5, 6, 7, and 9) and DA (8, 10, 11, 12, and 13). By contrast, BPC1 bound to the major groove of dsRNA and the conformation of BPC1 was obviously twisted (Fig. 2i). The energy of BPC1 can still return to the ground state through non radiative decays. The analysis implied that the BPC1–DNA complex could effectively restrain the free rotation of BPC1*via* the hydrophobic interactions and Van der Waals forces, thereby maximizing the energy of radiative decays and lighting up the BPC1 fluorescence in aqueous solution.

### 2.6 Cell imaging

To determine the ability of the four dyes to stain the cell nucleus, we investigated the signal distribution on MCF-7 cells exposed to these dyes and quantified the ratio of fluorescence intensity between the nucleus and cytoplasm (Fig. S9†). Interesting, only BPC1 has a high nuclear to cytoplasm ratio (7.4), enabling clear staining of the cell nucleus. To further clarify the relationship between the structures of the dyes and permeability, we determined the oil–water partition coefficient (*C* log *P*) of the four dyes (Table S7†). The data showed that the *C* log *P* value of BPC1 was 2.73, within the range of 1.84 (QPC1, QPC2) to 2.9 (BPC2), indicating that the outstanding cellular membrane permeability of BPC1 was due to an appropriate lipophilic balance.

**Fig. 3:**
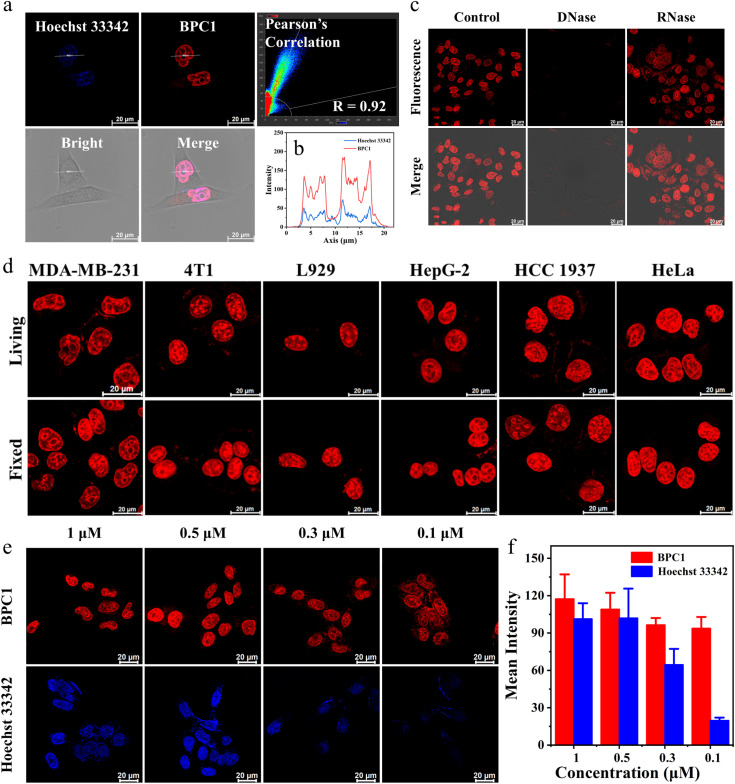
(a) Confocal imaging of living MCF-7 cells co-stained with BPC1 (0.5 μM) and Hoechst 33342 (1 μM). Signal distribution and Pearson's correlation were obtained from LAS AF. (b) Curve chart: intensity correlation of BPC1 and Hoechst 33342. (c) Confocal imaging of fixed MCF-7 cells co-stained with BPC1 (0.5 μM) following treatment with DNase I (100 U mL^−1^) or RNase A (100 μg mL^−1^), respectively. (d) Confocal imaging of MDA-MB-231, 4T1, L929, HepG-2, HCC 1937 and HeLa cells stained with BPC1 (0.5 μM). (e) Confocal imaging of MCF-7 cells stained with different doses of BPC1 or Hoechst 33342. (f) Statistical chart of mean fluorescence intensity. Scale bar: 20 μm.

Then, we performed colocalization analyses on cells exposed to BPC1 and Hoechst 33342 (a commonly used commercial nuclear dye). These cells were incubated with 0.5 μM BPC1 for 30 minutes, allowing for sufficient uptake of BPC1 within the cells. Subsequently, they were stained with 1 μM Hoechst 33342 for an additional 30 minutes and observed using Confocal Laser Scanning Microscopy (CLSM). As demonstrated in Fig. 3a and S10,†BPC1 (red) and Hoechst 33342 (blue) could efficiently reach the nucleus. The Pearson Correlation Coefficient (PCC) between the fluorescence signals (red and blue) was calculated to be 0.92 and 0.94 in the MCF-7 and MCF-10A cells, respectively. These suggested that BPC1 has the capability to selectively target the cell nucleus, offering potential for specific labeling of cell nucleus DNA at the cellular level. Notably, the curve chart demonstrated that the fluorescence intensity line of BPC1 (red line) exhibited a smoother pattern than that of Hoechst 33342 (blue line) (Fig. 3b), which suggested a significantly higher signal-to-noise ratio for BPC1 imaging in comparison to Hoechst 33342.

Then, fixed MCF-7 and MCF-10A cells were stained with BPC1 and Hoechst 33342. The fluorescence analysis in Fig. S10† revealed a substantial overlap between the fluorescence of BPC1 and the blue fluorescence of Hoechst 33342 in fixed MCF-7 and MCF-10A cells, indicating BPC1's outstanding localization ability for fixed cells.

Furthermore, to confirm that the fluorescence signal in cells specifically arises from BPC1 binding to nuclear DNA, MCF-7 cells were fixed and fluorescence of BPC1 was measured pre- and post-treatment with DNase I and RNase A (Fig. 3c). As expected, the fluorescence intensity of BPC1 was significantly reduced after treatment with DNase I, and the fluorescence intensity had hardly changed after treatment with RNase A. The results confirmed that the fluorescence intensity primarily originated from BPC1's interaction with nuclear DNA in cells.

To assess the universality of BPC1's capability for labeling the cell nucleus, more types of cells were stained with BPC1. The results (Fig. 3d) indicated that BPC1 had remarkable nuclear labeling capability across various cell types. It was noteworthy that chromatin distribution in most cells tends to be dispersed and located near the nuclear membrane. In contrast, the chromatin in the L929 cell nucleus was concentrated near the nuclear membrane, while in 4T1 cells, it was centralized around the nucleus's core. The findings suggested that in addition to labeling the cell nucleus, BPC1's fluorescence signal can partly reflect the distribution of chromatin within the nucleus.^41–43^

**Fig. 4:**
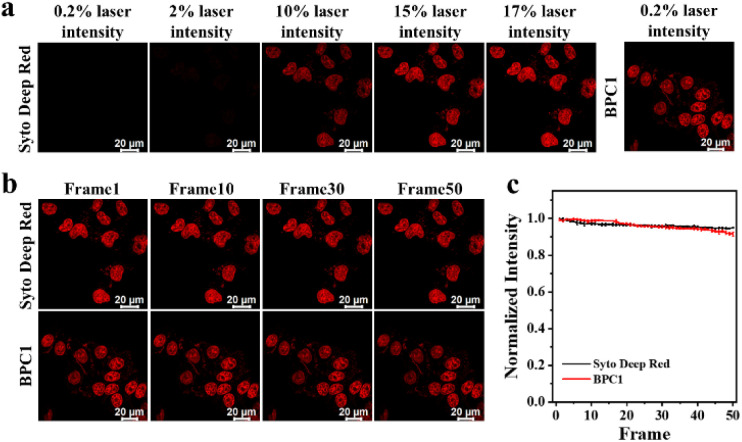
(a) Confocal imaging of MCF-7 cells co-stained with BPC1 (0.5 μM) or SYTO Deep Red respectively at varying degrees of laser intensity. (b) Consecutive confocal imaging and (c) quantitative analysis of MCF-7 cells co-stained with BPC1 (0.5 μM) or SYTO Deep Red respectively. Scale bar: 20 μm.

Since a lower dose not only reduces costs but also demonstrates higher biocompatibility, we next sought to validate the image quality after reducing the dye dosage. As shown in Fig. 3e and f, it still effectively labels the cell nucleus even when the concentration of BPC1 was as low as 100 nM. Conversely, the cell nucleus could not be clearly observed at concentrations below 300 nM after incubation with Hoechst 33342. MTT experiments (Fig. S11 and S12†) showed that cell survival exceeded 90% after co-incubating with BPC1 for 12 h, 24 h or even 48 h at working concentrations (100 nM), indicating that BPC1 has good biocompatibility and can't affect cell proliferation. Furthermore, we compared BPC1 with the commercial red DNA labeling dye SYTO Deep Red (Fig. 4a). It was observed that with the same laser intensity (0.2% rated power, 0.8 μW), BPC1 effectively labeled the cell nucleus, greatly reducing light-induced damage to the cells. However there was no observable fluorescence signal for SYTO Deep Red until a further increase in laser intensity to 17% (68 μW). After fifty consecutive photos were captured in the designated field of view with their corresponding matched powers (Fig. 4b and c), the fluorescence intensity showed no significant decrease, suggesting that BPC1 possesses anti-photobleaching properties and meets the requirements for cell biology research. Subsequently, we performed continuous imaging of individual cells using BPC1 (ESI Movie†). The results demonstrate that BPC1 effectively anchors the cell nucleus without causing significant staining in other cellular regions over a 5-hour-long imaging period. Further imaging experiments confirmed the ability of BPC1 to track the cell division process (Fig. S13†), highlighting its efficacy as a fluorescent marker for observing dynamic structural changes in the cell nucleus.

**Fig. 5:**
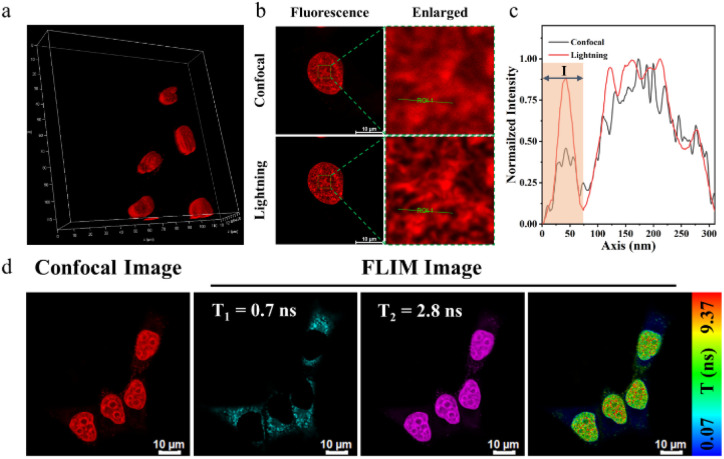
(a) Confocal 3D imaging of the nucleus using BPC1 (0.5 μM). (b) Confocal lightning imaging of the nucleus using BPC1 (0.5 μM) from LAS AF. Scale bar: 10 μm. (c) Intensity correlation of confocal and lightning imaging. (d) FLIM images of the nucleus recorded using BPC1 (0.5 μM). Scale bar: 10 μm.

Fluorescence dye-based imaging technologies such as 3D imaging, super-resolution fluorescence imaging, and Fluorescence Lifetime Imaging Microscopy (FLIM), have gained notable advances in recent years.^44,45^ We first performed 3D reconstruction to get clear 3D confocal images (Fig. 5a). The unfolded MCF-7 cell nucleus presented an elliptical spherical shape. These indicated that BPC1 can spatially analyse subcellular structures in various directions. Next, we assessed the performance of BPC1 in super-resolution fluorescence imaging using Leica Lightning Technology (Fig. 5b). Upon local magnification of the region of interest, the cell nucleus chromatin in the confocal imaging appeared blurry, making it difficult to distinguish between adjacent chromatin. In contrast, the chromatin in lightning super-resolution imaging appeared clear, displaying the shape of the chromatin, underscoring BPC1 as a potent tool for observing the intricate structure of the cell nucleus by super-resolution imaging.

Compared to traditional imaging methods based on fluorescence intensity, the fluorescence lifetime is unaffected by changes in excitation light intensity and fluorescence molecule concentration, but it is influenced by the microenvironment surrounding the molecule, including viscosity, polarity, temperature, and pH. This makes the fluorescence lifetime more suitable for the quantitative imaging of microenvironments in biological systems. Given the property that the fluorescence lifetime of BPC1 increases after binding to DNA, we utilized FLIM technology to monitor the signal distribution of BPC1 in living cells. As shown in Fig. 5d, we separated the short lifetime (0.7 ns) generated by non-specific dye retention from the long lifetime (2.8 ns) generated by DNA specific binding, achieving more accurate imaging of nuclear DNA. The above cell experimental results demonstrated that BPC1 is a powerful tool for observing the structure, size, and spatial distribution of the cell nucleus.

### 2.7 Human peripheral blood sample analyses

**Fig. 6:**
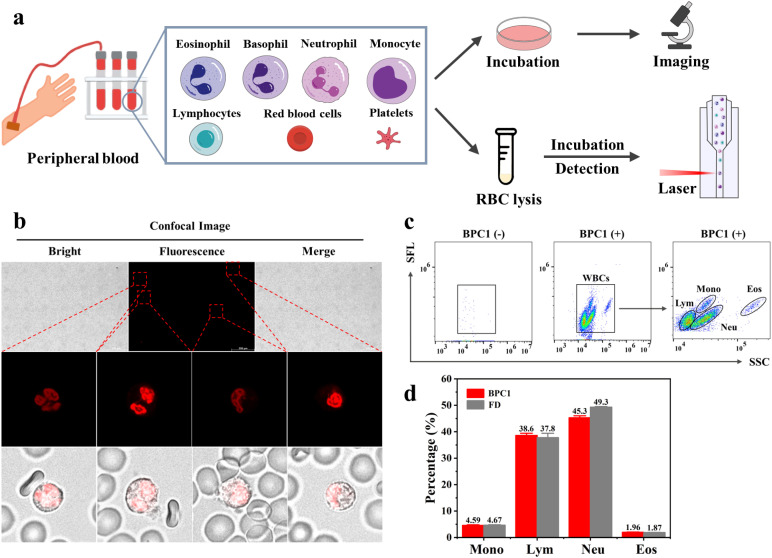
(a) Workflow for the peripheral blood sample preparation. (b) Confocal imaging of peripheral blood cells stained with BPC1 (0.5 μM): splicing diagram and the local magnification diagram. (c) Flow cytometry dot-plot image of staining with BPC1 (5 μM) on peripheral blood cells. (d) The quantified percentage of different kinds of WBCs.

Peripheral blood contains various types of blood cells, such as White Blood Cells (WBCs), Red Blood Cells (RBCs), and Platelets (PLTs).^46^ Specifically, red blood cells and platelets lack the nucleus, while white blood cells consist of five main types (monocytes, lymphocytes, neutrophils, eosinophils, and basophils) exhibiting variation in nucleic acid content and cell morphology.^47^ For instance, neutrophils possess rod-shaped or lobed cell nuclei, whereas lymphocytes have elliptical cell nuclei. Based on BPC1's excellent performance for the selective labeling of nuclear DNA, we further explored whether BPC1 can be applied for leukocytes sorting with real blood samples from a hospital. As shown in Fig. 6b, we successfully captured the rod-shaped, bilobed, and trilobed cell nuclei of neutrophils by using CLSM imaging after staining with BPC1, as well as the elliptical cell nuclei of lymphocytes. Furthermore, granulocytes consist of neutrophils, eosinophils, and basophils with similar size and complexity, which make it difficult to distinguish each other in Forward Scatter (FSC) *vs.* Side Scatter (SSC) by flow cytometry. Given the super sensitivity to DNA, the fluorescence intensity can represent the amount of DNA in the cells. Therefore, we used flow cytometry to achieve leukocyte subtyping. First, the cells were treated with red blood cell lysis buffer (F-LD, selectively lyses red blood cells and keeps the original characteristics of the leukocytes as much as possible) for 5 minutes, and then BPC1 (5 μM) was directly added to the cells and incubated for 1 minute. Four parts could be remarkably separated including monocytes, lymphocytes, neutrophils, and eosinophils by collecting SSC *vs.* Side Fluorescence (SFL) using a flow cytometer (Fig. 6c and d). Interestingly, these results confirmed that monocytes and eosinophils had a higher DNA content than lymphocytes and neutrophils by comparison of the SFL axis fluorescence intensity. Moreover, we conducted the same test using commercial white blood cell classification dyes (FD, Automatic Hematology Analyzer F-FD Fluorescent Dye, can divide white blood cells into different groups based on fluorescence intensity). According to the flow cytometry dot-plot images and statistical results shown in Fig. S14† and 6d, the count percentage of four kinds of WBCs in the BPC1 group was similar to that of the FD group. And the most of the coefficients of variation (CV) for BPC1 and FD were below 10% (Tables S8 and S9†), revealing that BPC1 can achieve the classification of WBCs based on the quantification of DNA content. These results indicated that DNA marker BPC1 also showed great potential to facilitate blood cell classification and counting *via* flow cytometry.

## 3 Conclusions

In summary, a series of new asymmetric cyanine dyes with nucleic acid recognition properties have been designed and synthesized by incorporating various nitrogen-containing heterocycles. These dyes demonstrated high selectivity for DNA under long-wavelength excitation (550 nm to 700 nm). Notably, among these dyes, BPC1 exhibited the highest fluorescence brightness compared to existing DNA labeling dyes, offering excellent DNA specificity and cell permeability. BPC1 can label the cell nucleus at extremely low doses (100 nM) and laser power (0.8 μW), making it suitable for real-time and high-fidelity imaging. Moreover, our study demonstrated the adaptability of BPC1 to various microscopy techniques, including 3D confocal imaging, super-resolution imaging, and fluorescence lifetime imaging. In addition to these remarkable properties, BPC1 was utilized for staining clinical blood samples, exhibiting excellent blood cell classification and counting ability by labeling the nuclei of WBCs, highlighting its significant potential as a clinical diagnostic tool, particularly effective in conjunction with existing blood cell analyzers. Overall, we foresee substantial commercial prospects for BPC1 and its potential to serve as a powerful tool in life sciences and cell biology research.

## Data availability

The data supporting this article have been included as part of the ESI.†

## Author contributions

Z. P. Li designed and performed all the experiments and wrote the manuscript. Q. C. Yao participated in calculation and data analysis. Z. Liu, D. Yu and W. Y. Ma participated in some experiments including *in vitro* tests and cell experiments. J. L. Fan directed the whole process in this work, guided the writing and revised the manuscript. C. Y. Zhang, J. L. Fan, and X. J. Peng offered constructive suggestions on the improvement of this work and provided financial support.

## Conflicts of interest

The authors declare that they have no known competing financial interests or personal relationships that could have appeared to influence the work reported in this paper.

## Supplementary Material

SC-OLF-D4SC04848B-s001

SC-OLF-D4SC04848B-s002
