# Supplementary material for: Next-generation red ultra-bright fluorescent dyes for nuclear imaging and peripheral blood leukocytes sorting
Source: Chem Sci. 2024 Sep 27;15(42):17524–32. doi: 10.1039/d4sc04848b (PMC11451967; doi:10.1039/d4sc04848b)
Supplement: SC-015-D4SC04848B-s001 [file SC-015-D4SC04848B-s001.pdf]

Supplementary Information (SI) for Chemical Science.
This journal is © The Royal Society of Chemistry 2024

Supporting Information

Next-generation Red Ultra-bright Fluorescent Dyes for Nuclear Imaging and Peripheral Blood leukocytes Sorting

Zipeng Li, [a] *Zheng Liu,* [a] *Ding Yu,* [a] *Qichao Yao,* [a] *Wanying Ma,* [b] *Changyu Zhang,* [ab] *Jiangli Fan,* [*abc] *and Xiaojun Peng* [ac]

[a] State Key Laboratory of Fine Chemicals, Frontiers Science Center for Smart Materials Oriented Chemical Engineering, Dalian University of Technology, Dalian 116024, China. E-mail: fanjl@dlut.edu.cn.

[b] Ningbo Institute of Dalian University of Technology, Ningbo 315016, China.

[c] Liaoning Binhai Laboratory, Dalian 116023, China.

*Correspondence author. Prof. J. Fan. Email: fanjl@dlut.edu.cn.

1 Experimental Procedures

1.1 Materials and Reagents

All chemical reagents and solvents for synthesis and characterization were purchased from Aladdin, Energy Chemical, Bidepharm and Makclin and were used without further purification. Deoxyribonucleic acid from calf thymus (CT DNA, dsDNA), ribonucleic acid from torula yeast (yeast RNA, ssRNA), **Hoechst 33342** and 4% paraformaldehyde were purchased from Solarbio. PBS buffer was purchased from VivaCell. Oligonucleotides were purchased from Sangon Biotech. **SYTO Deep Red** was purchased from Thermo Fisher Scientific. Distilled water was used after passing through a water ultra-purification system. Fetal bovine serum (FBS) and penicillin/streptomycin were obtained from Biological Industries and Beijing Solarbio Science&Technology Co., Ltd. respectively. Dulbecco's Modified Eagle Medium (DMEM) and Mammary Epithelial cell Basal Medium (MEBM) were purchased from Beijing Solarbio Science&Technology Co., Ltd. DNase I and RNase A were purchased from Beyotime. The MCF-7, MDA-MB-231, 4T1, L929, HepG-2, HeLa and HCC 1937 cells were purchased from American Type Culture Collection. The peripheral blood samples from volunteers were obtained by Ningbo Medical Treatment Center Lihuili Hospital (Ningbo, China). These experiments were approved by the Medical Ethics Committee of Lihuili Hospital, Ningbo Medical Center (KY2024SL055-01), and informed consent was obtained.

1.2 Instruments

UV-vis spectra and fluorescent spectra were conducted on a Fluorescence and Absorbance Spectrometer (Duetta, HORIBA). ^{1}H and ^{13}C NMR spectra were recorded on a Bruker Ascend^{TM} 400 spectrometer using TMS as the internal reference. High Resolution Mass Spectrometric (HR-MS) data were obtained on a Quadrupole Time-of-Flight Liquid Chromatograph Mass Spectrometer (LCMS-9030, Shimadzu). Fluorescence quantum yield (Φ_F) was obtained with the HAMAMATSU absolute

fluorescence quantum yield spectrometer (Serial No. C11347). The fluorescent lifetimes were measured using an Edinburgh FLS1000 spectrofluorometer with a time-correlated single photon counting technique. The cell imaging experiments were measured by Leica Confocal Laser Scanning Microscope (STELLARIS 5).

1.3 Synthesis and Characterization

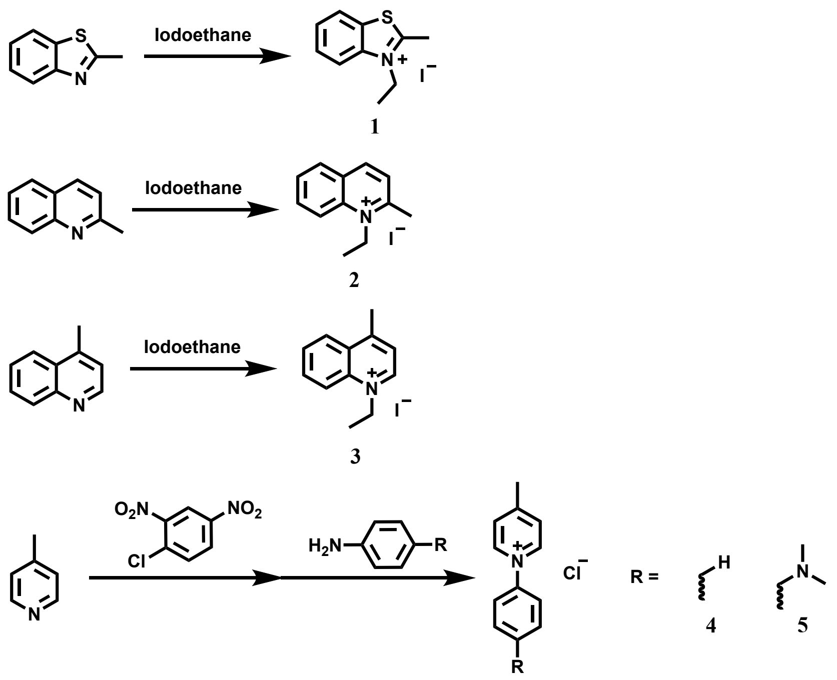

Scheme S1. Synthetic routes of compounds **1**, **2**, **3**, **4**, and **5**.

Synthesis of **compound 1**:

An oven-dried 100 mL round-bottom flask was charged with 2-Methylbenzothiazole (2.00 g, 13.40 mmol, 1.0 equiv.) and Iodoethane (2.51 g, 16.08 mmol, 1.2 equiv.). The reaction mixture was stirred for 6 hours at 110 °C, then cooled to room temperature. At this stage, ethyl acetate (50 mL) was added and stirring was continued for another 1 h. The resulting suspension was filtered and washed with ethyl acetate (3 × 15 mL), and dried. The resulting white solid was used without any further purification (1.03 g, 25%).

Synthesis of **compound 2**:

An oven-dried 100 mL round-bottom flask was charged with 2-Methylpuinoiline (3.00 g, 20.95 mmol, 1.0 equiv.) and Iodoethane (9.8 g, 62.85 mmol, 3.0 equiv.). The reaction

mixture was stirred for 10 hours at 110 ℃, then cooled to room temperature. At this stage, ethyl acetate (50 mL) was added and stirring was continued for another 1 h. The resulting suspension was filtered and washed with ethyl acetate (3 × 15 mL), and dried. The resulting yellow solid was used without any further purification (2.50 g, 40%).

Synthesis of **compound 3**:

An oven-dried 100 mL round-bottom flask was charged with 4-Methylpuinoiline (5.00 g, 34.92 mmol, 1.0 equiv.) and Iodoethane (7.62 g, 48.89 mmol, 1.4 equiv.). The reaction mixture was stirred for 3 hours at 80 ℃, then cooled to room temperature. At this stage, acetonitrile (50 mL) was added and stirring was continued for another 1 h. The resulting suspension was filtered and washed with acetonitrile (3 × 15 mL), and dried. The resulting yellow solid was used without any further purification (10.35 g, 99%).

Compound 4 and **5** was synthesized according to the previous literature[1]

Step 1. An oven-dried 100 mL round-bottom flask was charged with 2,4-Dinitrochlorobenzene (10.13 g, 50.00 mmol, 1.0 equiv.), 4-Methylpyridine (5.12 g, 55.00 mmol, 1.1 equiv.) and 25 mL anhydrous ethanol. The reaction mixture was stirred for 3 hours at 85 ℃, then cooled to room temperature. At this stage, ether (100 mL) was added and stirring was continued for another 1 h. The resulting suspension was filtered and washed with dichloromethane (3 × 50 mL), and dried. The resulting black solid was used without any further purification (9.60 g, 65%).

Step 2.

Synthesis of **compound 4**:

Under nitrogen atmosphere, a 100-mL three-necked flask was charged with the **intermediate** from previous step (3.00 g, 10.15 mmol, 1.0 equiv.) and 20 mL anhydrous ethanol. The reaction mixture was heated to 85 ℃, then the solution of aniline (1.89 g, 20.29 mmol, 2.0 equiv.) in anhydrous ethanol (10 mL) was added. And stirring was continued for 5 hours at 85 ℃. Then cooled to room temperature and removed the solvent. The crude reaction mixture was purified on silica gel column chromatography to afford **compound 4** as black solid (0.79 g, 36%).

Synthesis of **compound 5**:

Under nitrogen atmosphere, a 100-mL three-necked flask was charged with the **intermediate** from previous step (3.00 g, 10.15 mmol, 1.0 equiv.) and 20 mL anhydrous ethanol. The reaction mixture was heated to 85 ℃, then the solution of N, N-Dimethylaniline (1.66 g, 12.18 mmol, 1.2 equiv.) in anhydrous ethanol (10 mL) was added. And stirring was continued for 4 hours at 85 ℃. Then cooled to room temperature and removed the solvent. The crude reaction mixture was purified on silica gel column chromatography to afford **compound 5** as black solid (1.6 g, 63%).

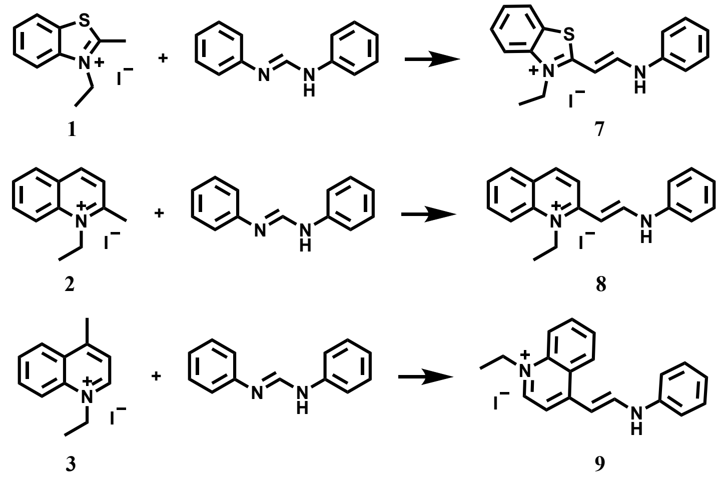

Scheme S2. Synthetic route of compounds **7**, **8**, and **9**.

Synthesis of compounds **7**, **8**, and **9**:

Compounds **7**, **8**, and **9** were obtained using similar synthetic procedure. Under nitrogen atmosphere, the mixture of quaternary ammonium salt derivatives (1 equiv.) and N, N'-Diphenylformamidine (1.2 eq) was stirred at 140 ℃ for 90 min and then cool to room temperature. The crude product obtained and crystallized by a mixed solvent of ethanol and ethyl acetate (yield: 75 - 90%).

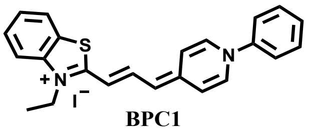

BPC1

Scheme S3. The structures of **BPC1**.

Synthesis of **BPC1**:

An oven-dried 25 mL round-bottom flask was charged with **compound 7** (400 mg, 0.979 mmol, 1.0 equiv.), **compound 4** (241 mg, 1.18 mmol, 1.2 equiv.), 0.8 mL N, N-Diisopropylethylamine, 0.4 mL acetic anhydride and 7.2 mL anhydrous pyridine. The

reaction mixture was stirred for 2 hours at room temperature. Then the reaction mixture was dropwise added to 20 mL of anhydrous ether solution (20 mL). The resulting suspension was filtered and purified on silica gel column chromatography to afford **BPC1** as purplish red solid (320 mg, 67%). ^{1}H NMR (400 MHz, DMSO-d_6) δ 8.49 (d, *J* = 7.1 Hz, 2H), 8.02-7.91 (m, 1H), 7.82 (d, *J* = 6.8 Hz, 1H), 7.75 (d, *J* = 7.8 Hz, 2H), 7.71 (s, 2H), 7.67 (t, *J* = 7.5 Hz, 2H), 7.60 (t, *J* = 7.3 Hz, 1H), 7.52 (d, *J* = 8.2 Hz, 1H), 7.45 (t, *J* = 8.4 Hz, 1H), 7.25 (t, *J* = 7.0 Hz, 1H), 6.28 (d, *J* = 6.4 Hz, 1H), 6.25 (d, *J* = 4.7 Hz, 1H), 4.24 (q, *J* = 7.1 Hz, 2H), 1.28 (t, *J* = 7.0 Hz, 3H). ^{13}C NMR (101 MHz, DMSO-d_6) δ 159.76, 153.21, 143.43, 142.67, 141.57, 140.71, 130.60, 130.05, 127.98, 125.04, 124.12, 123.91, 122.95, 119.43, 113.24, 112.26, 96.47, 40.77, 12.57. HR-MS: *m/z* calcd for $C_{23}H_{21}N_2S^+$ [M]$^+$: 357.1420, found: 357.1429.

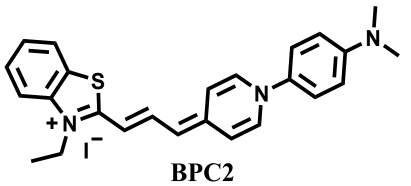

BPC2

Scheme S4. The structures of **BPC2**.

The **BPC2** was obtained using similar synthetic procedure to **BPC1**. Purplish red solid (yield: 56%). ^{1}H NMR (400 MHz, DMSO-d_6) δ 8.45 (d, *J* = 7.3 Hz, 2H), 7.85 (dd, *J* = 14.0, 12.0 Hz, 1H), 7.77 (d, *J* = 8.3 Hz, 1H), 7.70 (d, *J* = 6.8 Hz, 2H), 7.53 (d, *J* = 9.1 Hz, 2H), 7.45 (t, *J* = 6.1 Hz, 1H), 7.40 (d, *J* = 7.0 Hz, 1H), 7.21 (t, *J* = 6.7 Hz, 1H), 6.88 (d, *J* = 9.2 Hz, 2H), 6.27 (d, *J* = 14.0 Hz, 1H), 6.17 (d, *J* = 12.0 Hz, 1H), 4.19 (q, *J* = 7.1 Hz, 2H), 3.00 (s, 6H), 1.26 (t, *J* = 7.1 Hz, 3H). ^{13}C NMR (101 MHz, DMSO-d_6) δ 158.27, 152.39, 151.18, 142.11, 141.65, 140.64, 131.61, 127.83, 124.81, 124.26, 123.72, 122.81, 119.82, 113.60, 112.80, 111.84, 95.50, 40.51, 40.47, 12.42. HR-MS: *m/z* calcd for $C_{25}H_{26}N_3S^+$ [M]$^+$: 400.1842, found: 400.1855.

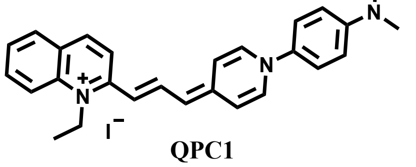

QPC1

Scheme S5. The structures of **QPC1**.

The **QPC1** was obtained using similar synthetic procedure to **BPC1**. Black solid (yield:

46%).^{1}H NMR (400 MHz, DMSO-d_6) δ 8.43 (t, *J* = 13.0 Hz, 1H), 8.35 (d, *J* = 7.1 Hz, 2H), 8.03 (d, *J* = 9.7 Hz, 1H), 7.75-7.55 (m, 6H), 7.51 (d, *J* = 9.1 Hz, 2H), 7.27 (t, *J* = 7.1 Hz, 1H), 6.87 (d, *J* = 9.2 Hz, 2H), 6.29 (d, *J* = 13.7 Hz, 1H), 6.00 (d, *J* = 12.2 Hz, 1H), 4.24 (t, *J* = 14.1 Hz, 2H), 2.99 (s, 6H), 1.33 (t, *J* = 7.0 Hz, 3H). ^{13}C NMR (101 MHz, DMSO-d_6) δ 152.38, 151.04, 149.49, 142.96, 140.03, 139.57, 133.17, 132.09, 131.71, 129.06, 124.27, 124.18, 123.75, 120.79, 119.24, 115.29, 114.13, 112.83, 102.20, 42.20, 40.48, 12.20. HR-MS: *m/z* calcd for $C_{27}H_{28}N_3^+$ [M]$^+$: 394.2278, found: 394.2294.

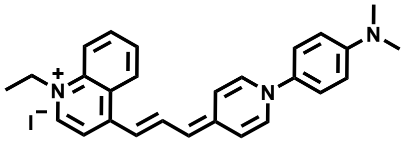

QPC2

Scheme S6. The structures of **QPC2**.

The **QPC2** was obtained using similar synthetic procedure to **BPC1**. Black solid (yield: 77%).^{1}H NMR (400 MHz, DMSO-d_6) δ 8.43 (t, *J* = 13.2 Hz, 1H), 8.34 (d, *J* = 7.0 Hz, 2H), 8.22 (d, *J* = 8.4 Hz, 1H), 7.74-7.66 (m, 5H), 7.52 (d, *J* = 9.0 Hz, 2H), 7.41 (dt, *J* = 8.2, 3.6 Hz, 1H), 7.36 (d, *J* = 7.6 Hz, 1H), 6.87 (d, *J* = 9.2 Hz, 2H), 6.66 (d, *J* = 12.5 Hz, 1H), 6.30 (d, *J* = 13.8 Hz, 1H), 4.26 (q, *J* = 7.0 Hz, 2H), 2.99 (s, 6H), 1.34 (t, *J* = 7.0 Hz, 3H). ^{13}C NMR (101 MHz, DMSO-d_6) δ 151.93, 151.04, 145.82, 141.54, 139.93, 138.65, 138.26, 132.20, 131.75, 125.15, 125.09, 124.15, 124.08, 119.44, 116.84, 115.09, 112.85, 107.57, 107.04, 47.73, 40.50, 14.70. HR-MS: *m/z* calcd for $C_{27}H_{28}N_3^+$ [M]$^+$: 394.2278, found: 394.2293.

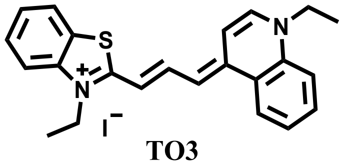

TO3

Scheme S7. The structures of **TO3**.

The **TO3** was obtained using similar synthetic procedure to **BPC1**. Golden yellow solid (yield: 94%).^{1}H NMR (400 MHz, DMSO-d_6) δ 8.48 (d, *J* = 7.4 Hz, 1H), 8.44 (d, *J* = 7.2 Hz, 1H), 8.16 (t, *J* = 12.8 Hz, 1H), 8.09 (d, *J* = 8.8 Hz, 1H), 7.96 (t, *J* = 7.2 Hz, 1H), 7.88 (t, *J* = 7.2 Hz, 2H), 7.71 (t, *J* = 7.5 Hz, 1H), 7.61 (d, *J* = 8.2 Hz, 1H), 7.49 (t, *J* = 7.2 Hz, 1H), 7.31 (t, *J* = 7.6 Hz, 1H), 7.14 (d, *J* = 13.3 Hz, 1H), 6.54 (d, *J* = 12.3 Hz, 1H), 4.61 (q, *J* = 7.1 Hz,

2H), 4.30 (q, J = 7.1 Hz, 2H), 1.45 (t, J = 7.1 Hz, 3H), 1.33 (t, J = 7.0 Hz, 3H). HR-MS: *m/z* calcd for $C_{23}H_{23}N_2S^+$ $[M]^+$: 359.1577, found: 359.1593.

1.4 In vitro characterizations

Absorption spectra

For absorption measurements, compounds of **Hoechst 33342** (5 µM), **TO3** (4 µM), **BPC1** (4 µM), **BPC2** (4 µM), **QPC1** (4 µM), or **QPC2** (4 µM) with or without nucleic acid (CT DNA: 100 µg/mL, yeast RNA: 100 µg/mL) were mixed in 10 mM PBS buffer (300 mM NaCl, pH 7.4) and absorbance spectra were recorded in the range of 300 nm-600 nm, 450 nm-750 nm or 500-800 nm.

Fluorescence spectra

Fluorescence spectra of **Hoechst 33342** (5 µM), **TO3** (4 µM), **BPC1** (4 µM), **BPC2** (4 µM), **QPC1** (4 µM), or **QPC2** (4 µM) with or without nucleic acid (CT DNA or yeast RNA) in 10 mM PBS buffer (300 mM NaCl, pH 7.4) were recorded with different excitation wavelength (**Hoechst 33342**: 350 nm, **TO3**: 630 nm, **BPC-1**: 600 nm, **BPC-2**: 605 nm, **QPC-1**: 635 nm, **QPC-2**: 690 nm).

For response kinetic studies, compounds of **BPC1**, **BPC2**, **QPC1**, or **QPC2** (4 µM) with or without nucleic acid (CT DNA: 100 µg/mL, yeast RNA: 100 µg/mL) were separately mixed in 10 mM PBS buffer (300 mM NaCl, pH 7.4) and fluorescence intensities at maximum emission wavelength were recorded in real time. Data points were taken every 1 s for 400 s.

For the quantum yield tests, we first used **Rhodamine B** as a standard to ensure the accuracy of the instrument (reference: 0.69 in ethanol, test: 0.67). Then, the quantum yields of **Hoechst 33342** and **BPC1** before and after binding to nucleic acid were measured separately by the HAMAMATSU absolute fluorescence quantum yield spectrometer (Serial No. C11347).

For the fluorescence lifetime tests, compounds of **BPC1**, **BPC2**, **QPC1**, or **QPC2** (4 µM) with or without nucleic acid (CT DNA: 100 µg/mL, yeast RNA: 100 µg/mL) were separately mixed in 10 mM PBS buffer (300 mM NaCl, pH 7.4) and fluorescence

lifetime decay curves were recorded. The fluorescence lifetime decay curves were fitted using the built-in software of the instrument.

For fluorescence concentration titration, compounds of **BPC1**, **BPC2**, **QPC1**, or **QPC2** (4 μM) were separately incubated with different concentrations of CT DNA or yeast RNA (0-100 μg/mL) and fluorescence spectra were obtained.

For fluorescence responses to varying concentrations of $ds(AT)_{20}$ DNA or $ds(GC)_{20}$ DNA, **BPC1** was incubated with different concentrations of DNAs and fluorescence spectra were obtained by microplate reader (Synergy H1, BIOTEK).

The association constants (Ka) of **BPC1**, **BPC2**, **QPC1** or **QPC2** to DNAs were calculated with Benesi-Hildebrand method. F_0 is the fluorescence intensity of dyes without DNAs, F is the fluorescence intensity measured with DNAs, F_{max} is the intensity of dyes in the presence of excessive DNAs. The values of $1/(F-F_0)$ were plotted against those for 1/[DNAs] and Ka is determined to be intercept/slope. For Job's plot, the fluorescence spectra for **BPC1** with different molar fractions of DNAs in Tris-HCl buffer (10 mM, pH 7.4, 50 mM KCl) were acquired. The total concentrations of DNAs and **BPC1** were fixed to 10 μM. Fluorescence intensities at 625 nm were plotted versus the molar fractions of DNAs.

For selectivity studies, **BPC1** (4 μM) was incubated with varying substances including blank, Na^+ (100 μM), K^+ (100 μM), PO_4^{3-} (100 μM), CO_3^{2-} (100 μM), CH_3COO^- (100 μM), $C_2O_4^{2-}$ (100 μM), H_2O_2 (100 μM), GSH (100 μM), glucose (100 μM), sucrose (100 μM), ATP (100 μM), L-Ala (100 μM), L-Leu (100 μM), L-Pro (100 μM), L-Trp (100 μM), L-Phe (100 μM), L-Gln (100 μM), L-Cys (100 μM), L-Asp (100 μM), L-Glu (100 μM), L-Lys (100 μM), L-His (100 μM), BSA (100 μM) and CT DNA (100 μg/mL). And fluorescence spectra were recorded respectively.

For oligonucleotides selectivity studies, **BPC1** (1 μM) was incubated with varying oligonucleotides (4 μM). And fluorescence spectra were recorded respectively.

CD spectra

Circular dichroism (CD) spectra were recorded on the Jasco J-810 spectrometer. A quartz cuvette with a 10 mm path length was used to record spectra over a wavelength range of 200-700 nm with a 1 nm step size. The CD-based titration assay

was performed at a fixed concentration of 0.5 mg/mL CT DNA or yeast RNA in 10 mM PBS buffer (300 mM NaCl, pH 7.4). The concentration-dependent changes in CD signal of **BPC1** was recorded. For CT DNA, the concentration of **BPC1** was 0, 0.05, 0.1, 0.15 and 0.2 mM. For yeast RNA, the concentration of **BPC1** was 0, 0.1, 0.2, 0.3, 0.4 and 0.5 mM.

Thermal melting assay

The UV-vis spectra based thermal melting assay was performed at a fixed concentration of **BPC1** (4 μM) in 10 mM PBS buffer (300 mM NaCl, pH 7.4) with or without 10 μg/mL CT DNA. Simultaneously, the same volume solution of DMSO that dissolved **BPC1** was also recorded to eliminate the interference of organic solvent. The UV-vis spectra data were recorded at intervals of 5 °C over a range of 50-95 °C. The final data of absorbance variation at 260 nm was analyzed to assess the thermal denaturation properties of CT DNA.

Computational details

For density functional theory calculations, all density functional theory calculations were performed using the Gaussian 09 program package. The ground state geometries were optimized at the B3LYP/6-31G* (d, p) level. The excitation state geometries were optimized at the B3LYP/TZVP (d, p) level. The solvent (water) effect was included in all calculations using the solvation model based on the density (PCM).

For molecular docking calculations, AutoDockTools platform were implemented to investigate the interaction between **BPC1** and nucleic acid (DNA or RNA). The crystal structures of nucleic acid from PDB database (DNA PDB ID: 3FDQ, RNA PDB ID: 6UGI) were selected as the research model. The final visualization of the calculation results was conducted using Pymol software.

For ClogP calculations, the ClogP of four dyes were calculated using ChemDraw Professional software.

Calculation of Average Lifetime

The photoluminescent decay curves were fitted and calculated according to the previous literature.[2]

The photoluminescent decay curves were fitted by the following double exponential

decay equation:

$$I(t) = I_0 + A_1 \exp(-t/\tau_1) + A_2 \exp(-t/\tau_2)$$

$$\tau = (A_1 \tau_1^2 + A_2 \tau_2^2) / (A_1 \tau_1 + A_2 \tau_2)$$

A_1 and A_2 were the frequency factors according to fitting model of transient photoluminescent decay curves. τ_1 and τ_2 were the lifetimes of the prompt and delayed decay components, respectively.

Calculation of the Radiative and Non-radiative Rate Constants

The radiative (k_r) and non-radiative (k_{nr}) rate constants were calculated by the following equation.[3, 4]

$$k_r = \Phi / \tau$$

$$k_{nr} = (1 - \Phi) / \tau$$

Φ_F: the fluorescence quantum yields. τ: the fluorescence lifetime.

1.5 Cellular assays

Cell Culture

MCF-7, MDA-MB-231, 4T1, L929, HepG-2, HeLa and HCC 1937 cells were cultured in Dulbecco's modified eagle's medium in high glucose (DMEM; Gibco) containing 10% fetal bovine serum (FBS; Gibco), and 1% penicillin/streptomycin (P/S; Gibco). MCF-10A cells were cultured in mammary epithelial cell basal medium (MEBM; Gibco) supplemented with 10% FBS, and 1% penicillin/streptomycin. All cells were cultured in a 5% CO_2 humidified incubator at 37 °C.

Confocal Fluorescence Imaging

MCF-7 and MCF-10A were grown in a 35-mm Petri dish with a 10-mm glass bottom well. After 12 h, the cells were washed with 1 mL × PBS for three times and incubated with **BPC1** in cell culture medium at 37°C before imaging. For living cells colocalization assays, MCF-7 or MCF-10A cells were incubated with **BPC1** (0.5 μM) for 30 min, and then **Hoechst 33342** (1 μM) was added and incubated for another 20 min. Confocal images were acquired using the following conditions: Excitation wavelength for **BPC1**: 600 nm, emission collection: 610 nm-710 nm; Excitation wavelength for **Hoechst**

33342: 405 nm, emission collection: 415 nm-515 nm. For fixed cells colocalization assays, the cells were fixed in cold ethanol for 30 min. After washing with PBS twice, the cells were incubated with **BPC1** (0.5 μM) for 15 min, and then **Hoechst 33342** (1 μM) was added and incubated for another 20 min. Confocal images were acquired using the following conditions: Excitation wavelength for **BPC1**: 600 nm, emission collection: 610 nm-710 nm; Excitation wavelength for **Hoechst 33342**: 405 nm, emission collection: 415 nm-515 nm.

To assess the universality of **BPC1**'s capability for labeling the cell nucleus, MDA-MB-231, 4T1, L929, HepG-2 and HCC 1937 cells were grown in a 35-mm Petri dish with a 10-mm glass bottom well. After 12 h, the cells were washed with 1 mL × PBS for three times. For living cells assays, cells were incubated with **BPC1** (0.5 μM) for 30 min, respectively. Confocal images were acquired. For fixed cells assays, the cells were fixed in cold ethanol for 15 min. After washing with PBS twice, the cells were incubated with **BPC1** (0.5 μM) for 30 min. Confocal images were acquired using the following conditions: Excitation wavelength for **BPC1**: 600 nm, emission collection: 610 nm-710 nm.

To test the ability for **BPC1** to stain DNA in cells, the MCF-7 cells were fixed in cold ethanol for 15 min. After washing with PBS twice, the cells were respectively incubated with different conditions for 2 h at 37 °C including Dnase I (100 U/mL), Rnase A (100 μg/ mL) or PBS, and washed again with PBS twice. Confocal images were acquired using the following conditions: Excitation wavelength for **BPC-1**: 600 nm, emission collection: 610 nm-710 nm.

To validate the image quality after reducing the dye dosage, MCF-7 cells were incubated with **BPC1** (0.1, 0.3, 0.5, 1 μM) or **Hoechst 33342** (0.1, 0.3, 0.5, 1 μM) for 30 min, then the fluorescence images were captured on confocal fluorescence microscope. Excitation wavelength for **BPC1**: 600 nm, emission collection: 610 nm-710 nm; Excitation wavelength for **Hoechst 33342**: 405 nm, emission collection: 415 nm-515 nm.

To compare the image quality between **BPC1** and **SYTO Deep Red**, MCF-7 cells were incubated with **BPC1** (0.5 μM) or **SYTO Deep Red** (1X) for 30 min. Keeping the other

parameters constant, only changing the laser intensity (0.2%, 2%, 10%, 15%, 17%). then the fluorescence images were captured on confocal fluorescence microscope. Excitation wavelength for **BPC1**: 600 nm, emission collection: 610 nm-710 nm; Excitation wavelength for **SYTO Deep Red**: 652 nm, emission collection: 662 nm-797 nm. For light stability testing, then fifty consecutive photos were captured in the designated field of view with their corresponding matched powers.

To evaluate the long-term imaging capability of **BPC1**, MCF-7 cells were incubated with **BPC1** (0.5 μM) for 30 min, then the fluorescence images were captured on confocal fluorescence microscope. After fixing the field of view, take a photo every 5 minutes for a duration of 325 minutes.

To explored the possibility of **BPC1** being compatible with various imaging modes, MCF-7 cells were incubated with **BPC1** (0.5 μM) for 30 min, then the fluorescence images were captured on confocal fluorescence microscope using the confocal 3D imaging module, Lightning imaging module, FLIM imaging module, respectively.

To image peripheral blood cells, peripheral blood cells were seeded in cell plates and then treated with **BPC1** (5.0 μM) at 37°C for 20 min. Confocal images were acquired using the following conditions: Excitation wavelength for **BPC1**: 600 nm, emission collection: 610 nm-710 nm.

1.6 Cytotoxicity assay

To validate the cytotoxicity of **BPC1**, MCF-7 cells (1 × 10^4 cells/well) in DMEM (supplemented with 10% FBS, 100 μL) were seeded in 96-well plate and incubated at 37 °C overnight. Then, the medium was changed and cells were treated with **BPC1** at a serial of concentrations (0, 0.1, 0.2, 0.4 and 0.8 μM) in DMEM (100 μL) at 37°C with 5% CO_2 humidified incubator for 12 h. Subsequently, the cell viability was determined by MTT (3-[4,5-dimethylthiazol-2-yl]-2,5 diphenyl tetrazolium bromide) assay based on the conversion of MTT into formazan crystals by living cells.

To further validate the cytotoxicity of **BPC1**, MCF-7 cells (1 × 10^4 cells/well) in DMEM (supplemented with 10% FBS, 100 μL) were seeded in 96-well plate and incubated at

37°C overnight. Then, the medium was changed and cells were treated with **BPC1** at a serial of concentrations (0, 0.1, 0.2, 0.3, 0.4, 0.5, 0.6, 0.7 and 0.8 μM) in DMEM (100 μL) at 37°C with 5% CO_2 humidified incubator for 24 h or 48 h. Subsequently, the cell viability was determined by MTT (3-[4,5-dimethylthiazol-2-yl]-2,5 diphenyl tetrazolium bromide) assay based on the conversion of MTT into formazan crystals by living cells.

1.7 Flow cytometric assay

The cells were treated with red blood cell lysis buffer (F-LD, selectively lyses red blood cells and keep the original characteristics of the leukocytes as much as possible) for 5 minutes, followed **BPC1** (5 μM) was directly added to the cells and incubated for 1 minute. For comparison, the cells were incubated with **FD** (commercial white blood cell classification dyes) and treated with the same conditions. Flow cytometry was performed using the Attune NxT Acoustic Focusing Cytometer (ThermoFisher). A 635 nm laser was used for excitation and fluorescence emission was collected with a 670/14 nm. 14,000 cells were analyzed for each sample and flow cytometry data were analyzed with FlowJoTM 10.

2. Supplemental Tables

Table S1. Photophysical Properties of commercial dyes and synthetic dyes.

	λ_{abs} [a] (nm)	λ_{em} [b] (nm)	I_{DNA}/I_{RNA}	ε_{max} [c] (M^{-1} cm^{-1})	Φ_F [d]	Brightness[e]
**Hoechst**	340	460		0.42×10^5	0.029	1218
**Hoechst** + RNA	346	474	11.8	0.39×10^5	0.068	2652
**Hoechst** + DNA	350	450		0.46×10^5	0.410	18860
**SytoDeep-Red**	660	668	3.54			
**SytoDeep-Red** + RNA	660	668				

**SytoDeep-Red** + DNA	658	668				
**TO3**	626	628		0.55×10^5	0.004	220
**TO3** + RNA	630	660	1.98	0.79×10^5	0.099	7821
**TO3** + DNA	630	652		0.81×10^5	0.137	11097
**BPC1**	580	621		1.22×10^5	0.012	1464
**BPC1** + RNA	584	632	7.09	0.95×10^5	0.126	11970
**BPC1** + DNA	600	627		1.33×10^5	0.540	**71820**
**BPC2**	580	628		0.54×10^5	0.035	1890
**BPC2** + RNA	580	646	11.10	0.38×10^5	0.112	4256
**BPC2** + DNA	605	637		0.72×10^5	0.566	40752
**QPC1**	615	634		0.77×10^5	0.003	231
**QPC1** + RNA	615	670	2.49	0.45×10^5	0.015	675
**QPC1** + DNA	635	665		0.86×10^5	0.028	2408
**QPC2**	665	690		0.57×10^5	0.005	285
**QPC2** + RNA	670	718	5.46	0.33×10^5	0.036	1188
**QPC2** + DNA	690	715		1.28×10^5	0.072	9216

[a] Absorption maxima. [b] Emission maxima. [c] Molar extinction coefficient. [d] Quantum yield. [e] Brightness is defined as the product of the molar extinction coefficient and the quantum yield ($\varepsilon \times \Phi$).

Table S2. Photophysical Properties of synthetic dyes.

	τ [a] (ns)	k_r [b] (s^{-1})	k_{nr} [c] (s^{-1})
**BPC1**			
**BPC1** + RNA	3.25	4.00×10^7	2.78×10^8
**BPC1** + DNA	3.47	1.55×10^8	1.32×10^8
**BPC2**			
**BPC2** + RNA	2.65	4.23×10^7	3.35×10^8
**BPC2** + DNA	3.08	1.84×10^8	1.41×10^8
**QPC1**			
**QPC1** + RNA	0.67	2.24×10^7	1.47×10^9
**QPC1** + DNA	0.42	6.71×10^7	2.33×10^9
**QPC2**			
**QPC2** + RNA	1.39	2.59×10^7	6.95×10^8
**QPC2** + DNA	0.99	7.29×10^7	9.40×10^8

[a] the fluorescence lifetime. [b] the radiative rate constants. [c] the non-radiative rate constants.

Table S3. Comparison of reported dyes for DNA labeling.

Structure	$\lambda_{ex}/\lambda_{em}$ (nm)	ε_{max} (M^{-1} cm^{-1})	Φ_F	Brightness	Intracellular distribution
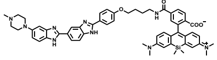 Nat. Commun., 2015, 6, 8497.	652/672	100000	0.17	17000	nucleus
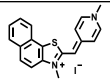 ACS Sens., 2022, 7, 469-476.	460/502	79000	0.164	13000	nucleus
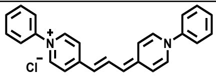 Nat. Commun., 2021, 12 (1), 2650.	654/666	150000	0.4	60000	nucleus
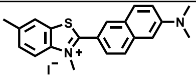 Analytical Chemistry, 2022, 94 (28), 10283-10290.	510/610	24228	0.35	8479	cytoplasm G-quadruplexes
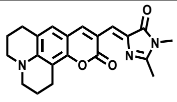 Chem. Sci., 2023, 14, 4538.	560/613	24700	0.47	11609	nucleus G-quadruplexes
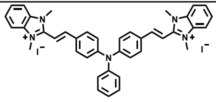 J. Am. Chem. Soc., 2013, 135 (34), 12697-706.	476/585	44400	0.54	23976	nucleus
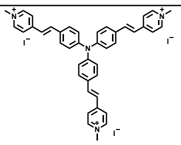 ChemBioChem, 2007, 8 (4), 424-33.	499/684	51000	0.021	1071	nucleus

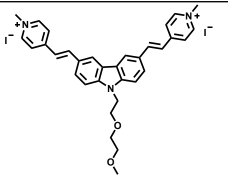 Org. Lett., 2010, 12, 2194.	451/548	49800	0.13	6474	cytoplasm
**This work (BPC1)**	**600/627**	133000	0.540	**71820**	**nucleus**

Table S4. Calculated $\lambda_{max,\ calc.}$ determined by the theoretical HOMO-LUMO gap (eV).

Compounds	λ_{abs} (nm)	λem (nm)	Transition orbit	HOMO /eV	LUMO/ eV	E_{gap}/eV
**BPC1**	566	586	H→L	-5.46	-2.85	2.61
**BPC2**	588	651	H→L	-5.32	-2.76	2.56
**QPC1**	611	680	H→L	-5.25	-2.79	2.46
**QPC2**	648	706	H→L	-5.17	-2.86	2.31

Table S5. Sequences for RNA and DNA oligonucleotides used in this study (ss: single strand, ds: double strand).

Structure	Names	Sequences(5'to3')
Duplex DNA extracted from calf thymus	calf thymus DNA	NA
All RNA extracted from yeast	yeast RNA	NA
DNA	$ds(A\text{-}T)_{20}$	AAAAAAAAAAAAAATTTTTTTTTTTTTT
	$ds(G\text{-}C)_{20}$	GGGGGGGGGGGGGCCCCCCCCCCCCC
	$dsDNA_{mix}$	CGATAAGCGCTTATCGAGTCGACTCGATAAGCG CTTATCG
	$ssDNA_{mix}$	ATGCTCAGACTGTCAGAGTC
	c-MYC	TGAGGGTGGGTAGGGTGGGTAA
	Tel26	AAAGGGTTAGGGTTAGGGTTAGGGAA
	hTelo_G4	TTAGGGTTAGGGTTAGGGTTAGGGTTA
	Mito 0.5-22	GGGGATGGCCATGGCTAGG
	Mito 0.5-22cs	CCTAGCCATGGCCATCCCC
	Mito	ACACTGATGCACTTTGTCTTCC
RNA	NC	UUGUACUACACAAAAGUACUG
	$ssRNA_{mix}$	AUGUACCUAGUCUGACCUAG

Table S6. The docking results for **BPC1** with DNA and RNA oligonucleotides.

oligonucleotides	Compounds	Docking Score (kcal/mol)
dsDNA<br>PDB 3FDQ	**BPC1**	-8.5
dsRNA<br>PDB 6UGI	**BPC1**	-8.0

Table S7. The ClogP of **BPC1**, **BPC2**, **QPC1** and **QPC2**.

Compounds	**BPC1**	**BPC2**	**QPC1**	**QPC2**
ClogP	2.73	2.90	1.84	1.84

Table S8. Statistical data of peripheral blood cells analysis with **BPC1** by flow cytometry.

**BPC1**	Replicate 1	Replicate 2	Replicate 3	M[a] ±SD[b]	CV%[c]
Mono (%, in WBCs)	4.60	4.79	4.39	4.59 ± 0.20	4.35
Lym (%, in WBCs)	39.50	37.90	38.50	38.60 ± 0.81	2.09
Neu (%, in WBCs)	46.10	44.60	45.20	45.30 ± 0.75	1.65
Eos (%, in WBCs)	2.00	2.05	1.83	1.96 ± 0.75	6.12

[a] Mean. [b] Standard deviation. [c] Coefficient of variation.

Table S9. Statistical data of peripheral blood cells analysis with **FD** by flow cytometry.

**FD**	Replicate 1	Replicate 2	Replicate 3	M ±SD	CV%
Mono (%, in WBCs)	4.80	4.72	4.50	4.67 ± 0.16	3.42
Lym (%, in WBCs)	36.00	38.40	39.10	37.80 ± 1.63	4.31
Neu (%, in WBCs)	49.50	49.20	49.10	49.30 ± 0.21	0.42
Eos (%, in WBCs)	1.90	1.91	1.80	1.87 ± 0.06	3.26

3. Supplemental Figures

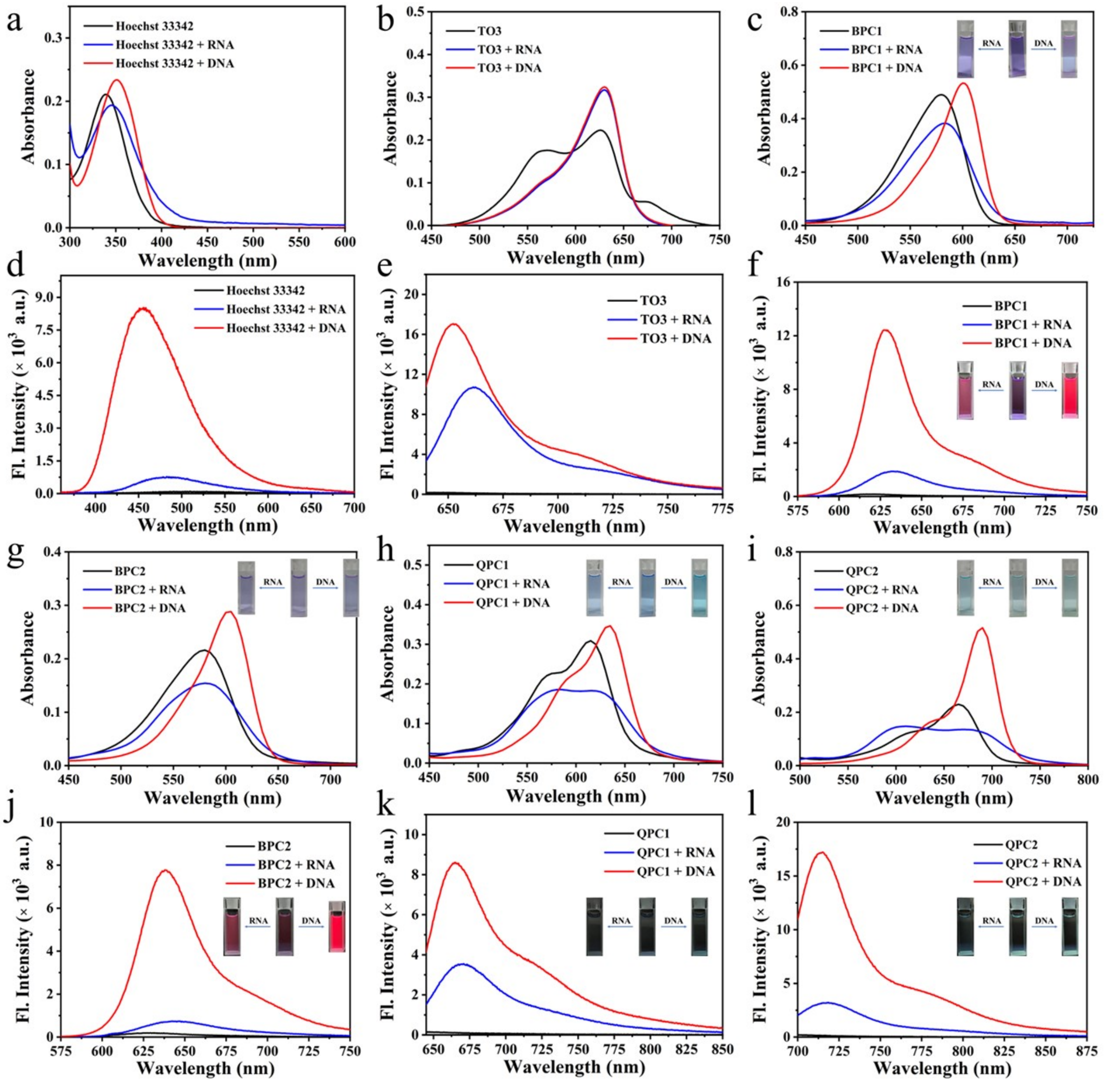

Figure S1. Absorbance spectra and fluorescence spectrum for commercial dyes **Hoechst 33342** (5 μM), **TO3** (4 μM), **BPC1** (4 μM), **BPC2** (4 μM), **QPC1** (4 μM) and **QPC2** (4 μM) in the absence or presence of nucleic acid (CT DNA: 100 μg/mL, yeast RNA: 100 μg/mL) in 10 mM PBS buffer (300 mM NaCl, pH 7.4).

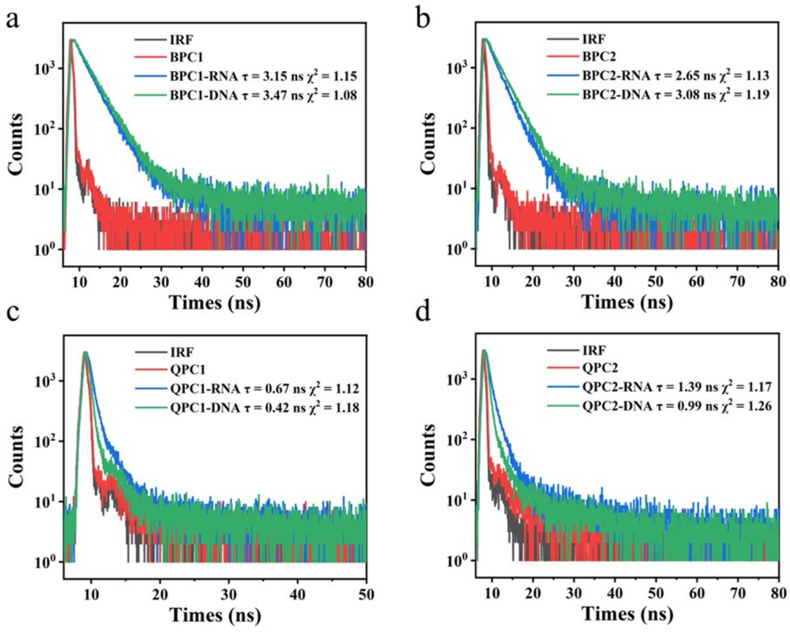

Figure S2. Lifetime changes of **BPC1**, **BPC2**, **QPC1** and **QPC2** (4 μM) in the absence or presence of nucleic acid (CT DNA: 100 μg/mL, yeast RNA: 100 μg/mL) in 10 mM PBS buffer (300 mM NaCl, pH 7.4).

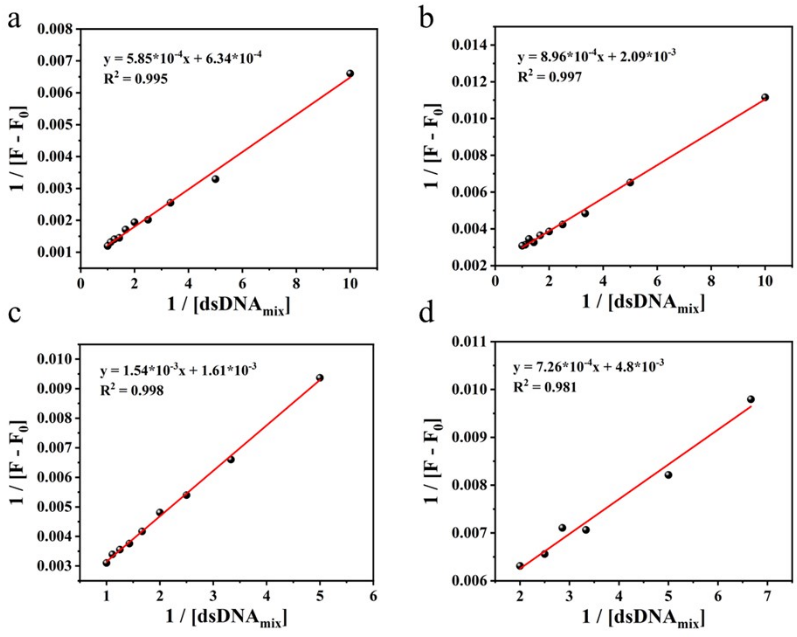

Figure S3. Benesi-Hildebrand plots of the reciprocal changes in fluorescence intensity of (a) **BPC1** at 625 nm, (b) **BPC2** at 637 nm, (c) **QPC1** at 665 nm and (d) **QPC2** at 715 nm as a function of the reciprocal $dsDNA_{mix}$.

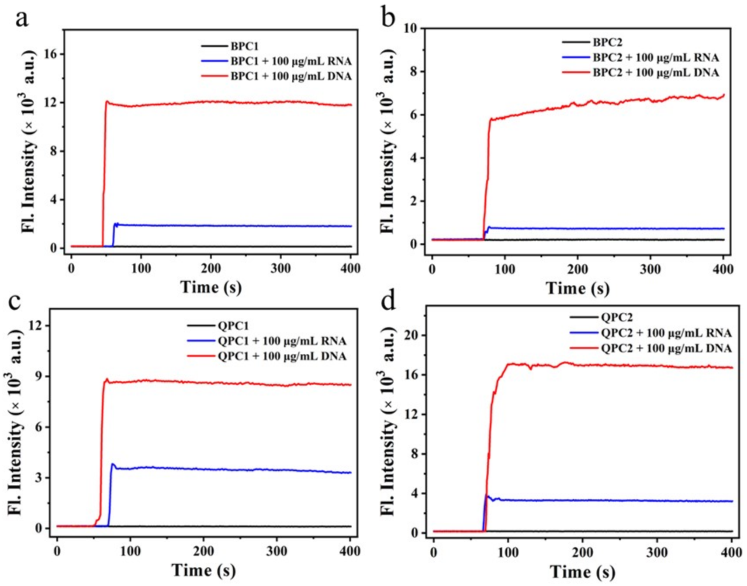

Figure S4. Fluorescence spectrum of response kinetic for **BPC1**, **BPC2**, **QPC1** and **QPC2** (4 μM) in the absence or presence of nucleic acid (CT DNA: 100 μg/mL, yeast RNA: 100 μg/mL) in 10 mM PBS buffer (300 mM NaCl, pH 7.4). Fluorescence intensities at maximum emission wavelength were recorded.

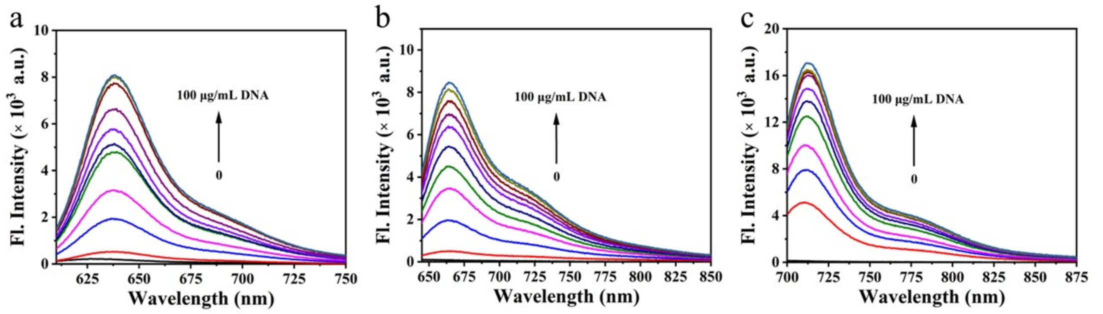

Figure S5. Fluorescence spectrum of concentration titration for **BPC2**, **QPC1** and **QPC2** (4 μM) with different concentrations of CT DNA (0-100 μg/mL).

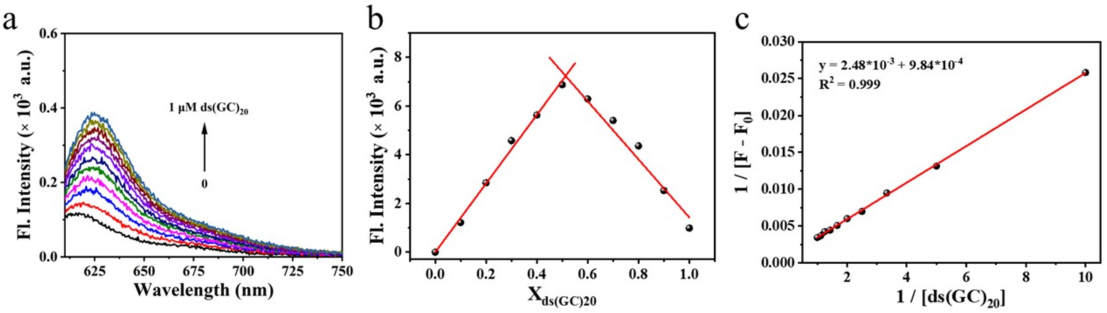

Figure S6. (a) Fluorescence spectrum of **BPC1** responses to varying concentrations of $ds(GC)_{20}$ DNA. (b) Job's plot for **BPC1** and $ds(GC)_{20}$ DNA. X-axis, mole fraction of

ds(GC)$_{20}$ DNA. The total concentrations for **BPC1** and ds(GC)$_{20}$ DNA were fixed to 10 μM. (c) Benesi-Hildebrand plots of the reciprocal changes in fluorescence intensity at 625 nm as a function of the reciprocal ds(GC)$_{20}$ DNA.

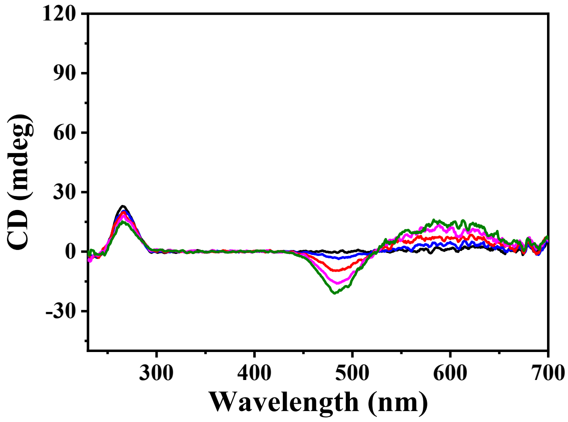

Figure S7. CD spectra of yeast RNA (0.5 mg/mL) to varying concentrations of **BPC1** (0-0.5 mM).

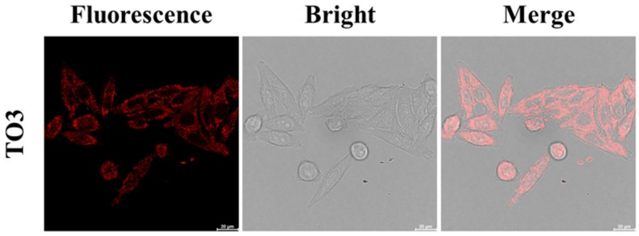

Figure S8. Confocal imaging of cells stained with **TO3** (0.5 μM). Scale bar: 20 μm.

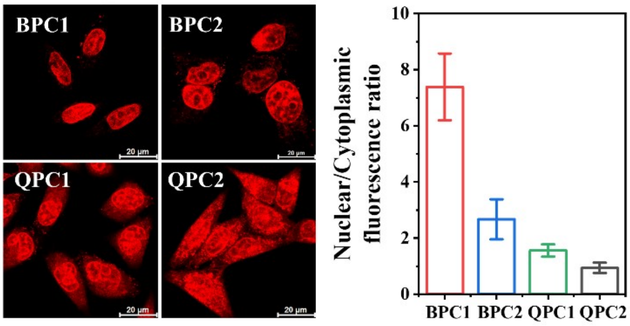

Figure S9. Confocal imaging of living MCF-7 cells stained with **BPC1** (1 μM), **BPC2** (1 μM), **QPC1** (1 μM) and **QPC2** (1 μM). Scale bar: 20 μm.

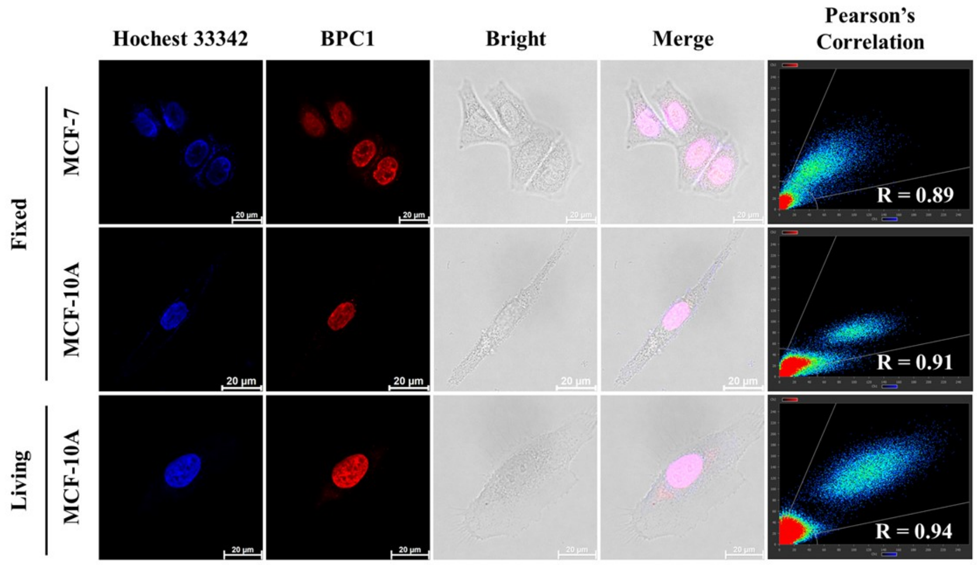

Figure S10. Confocal imaging of cells co-stained with **BPC1** (0.5 μM) and **Hoechst 33342**(1 μM). Scale bar: 20 μm.

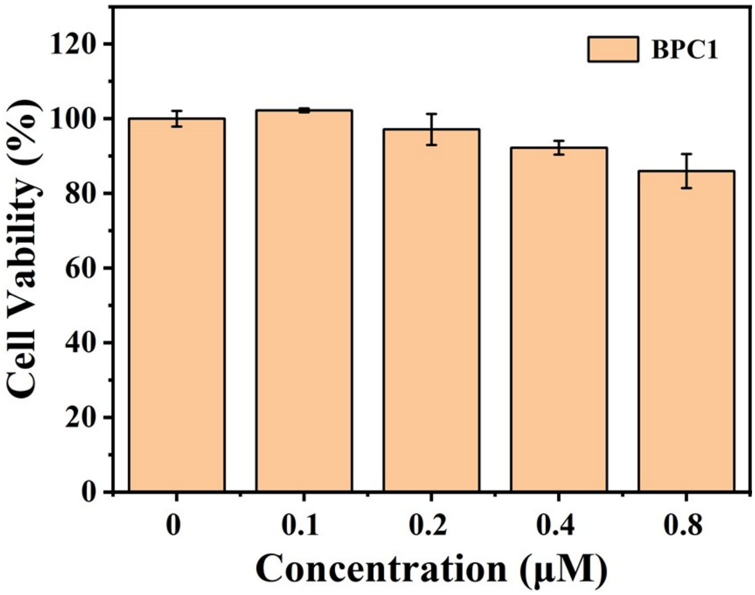

Figure S11. The cell viability test of MCF-7 cells co-incubating with **BPC1** for 12h.

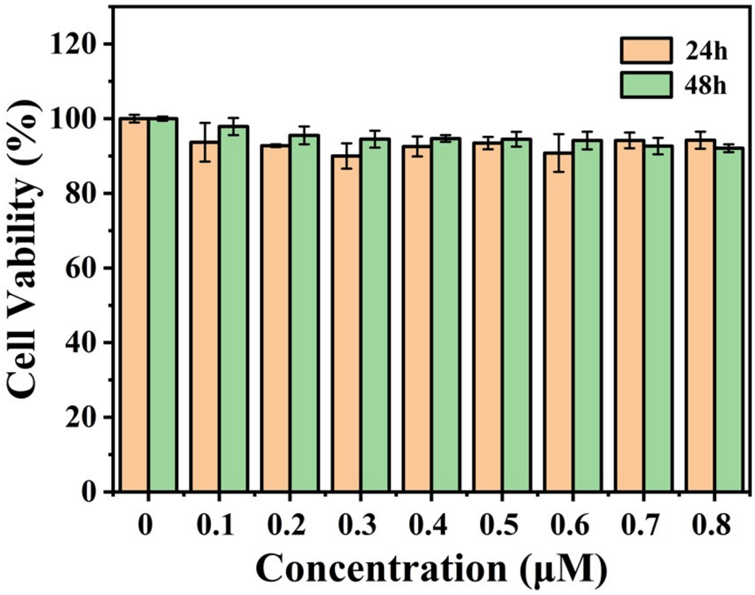

Figure S12. The cell viability test of MCF-7 cells co-incubating with **BPC1** for 24h or 48h.

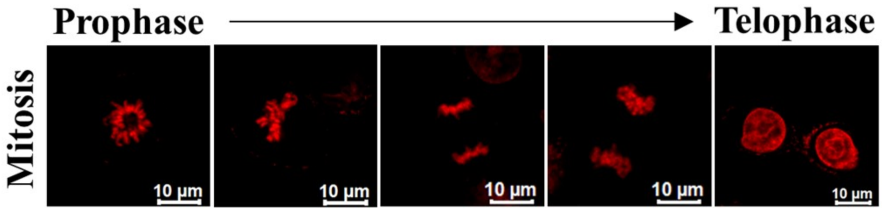

Figure S13. The monitoring of chromosomes behavior by **BPC1** during the cell cycle process.

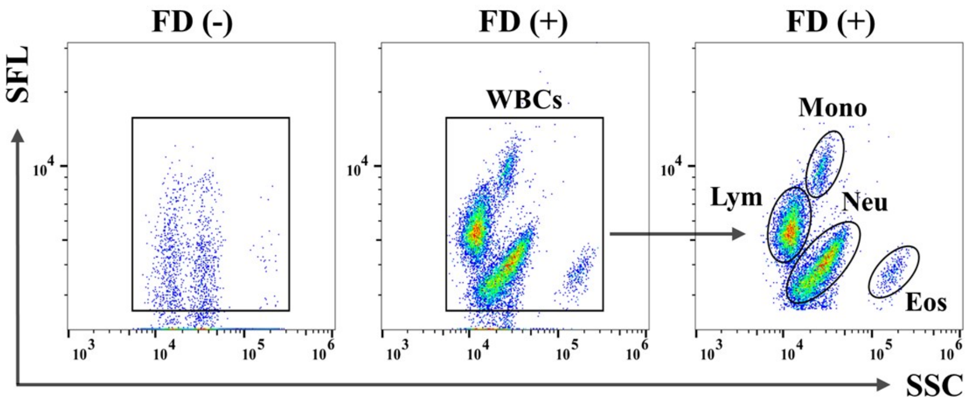

Figure S14. Flow cytometry dot-plot image of staining with **FD** on peripheral blood cells.

4. MS and NMR spectra

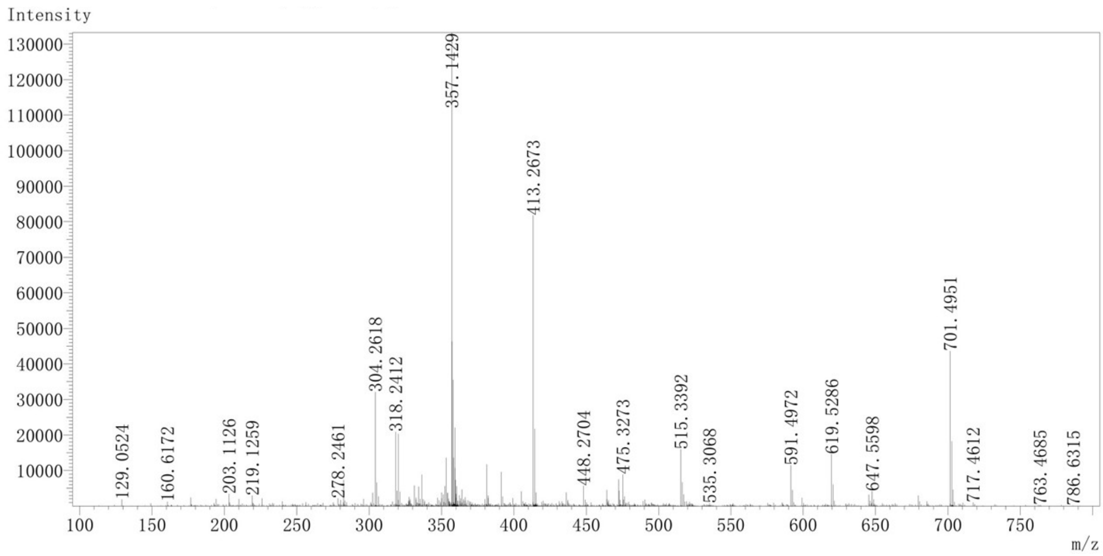

Figure S15. HR-MS spectrum of **BPC1**.

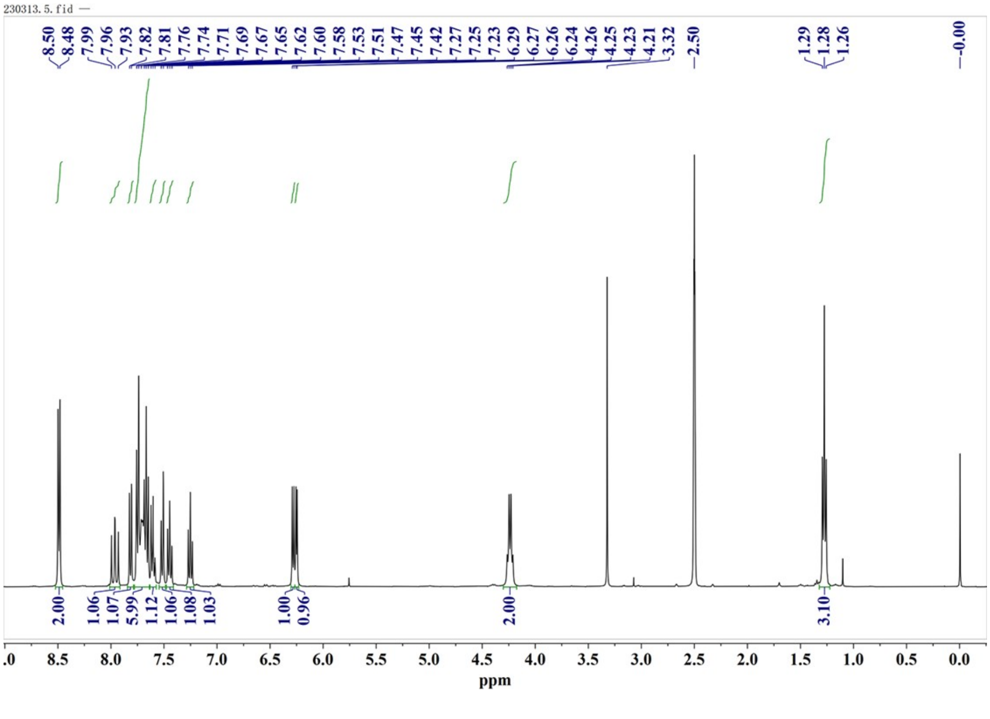

Figure S16. ^{1}H NMR spectrum of **BPC1** in DMSO-d_6.

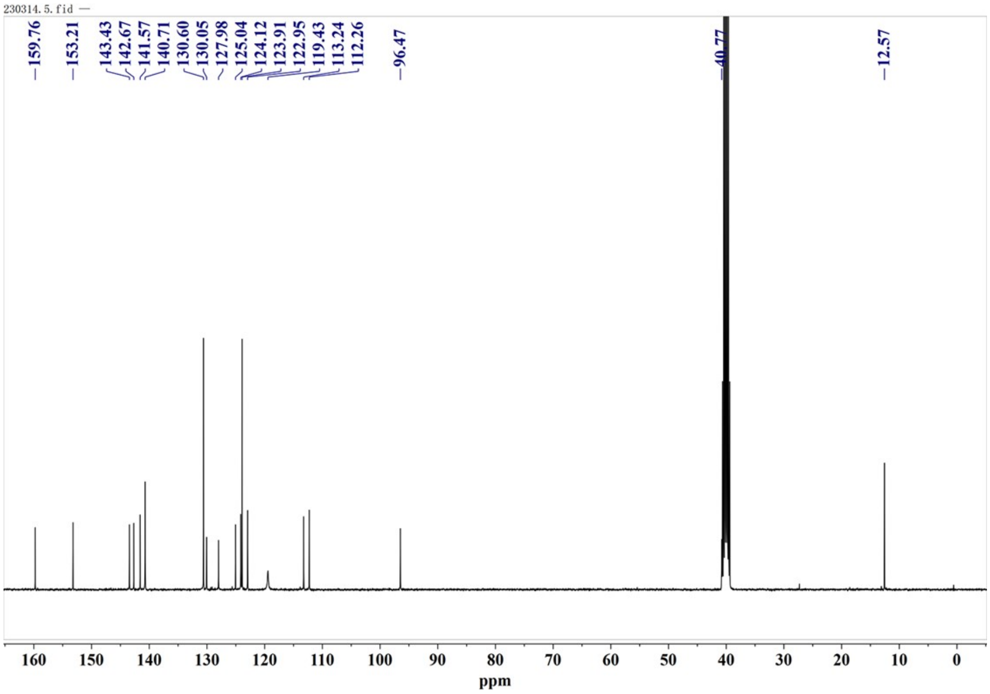

Figure S17. ^{13}C NMR spectrum of **BPC1** in DMSO-d_6.

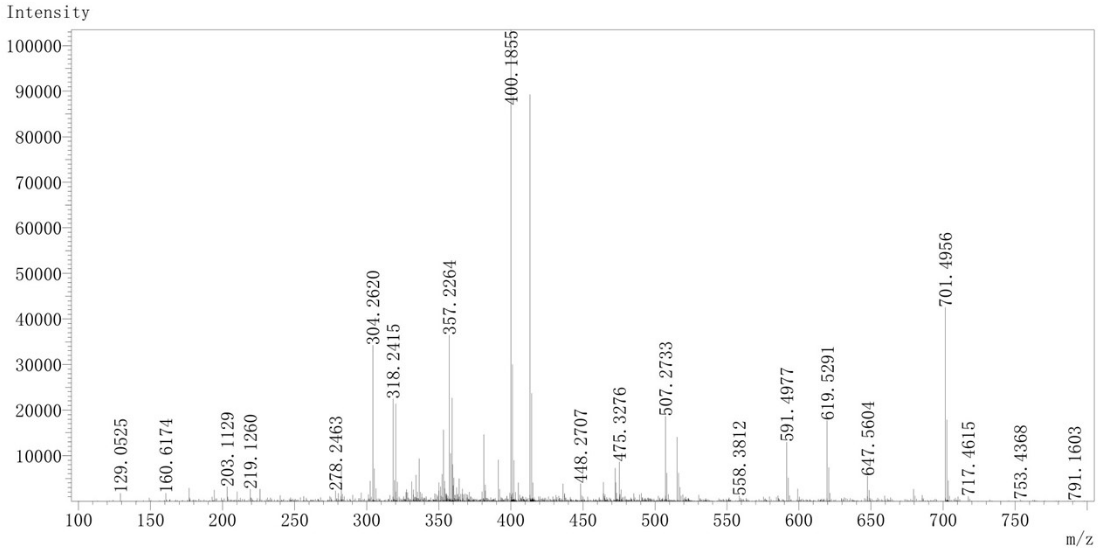

Figure S18. HR-MS spectrum of **BPC2**.

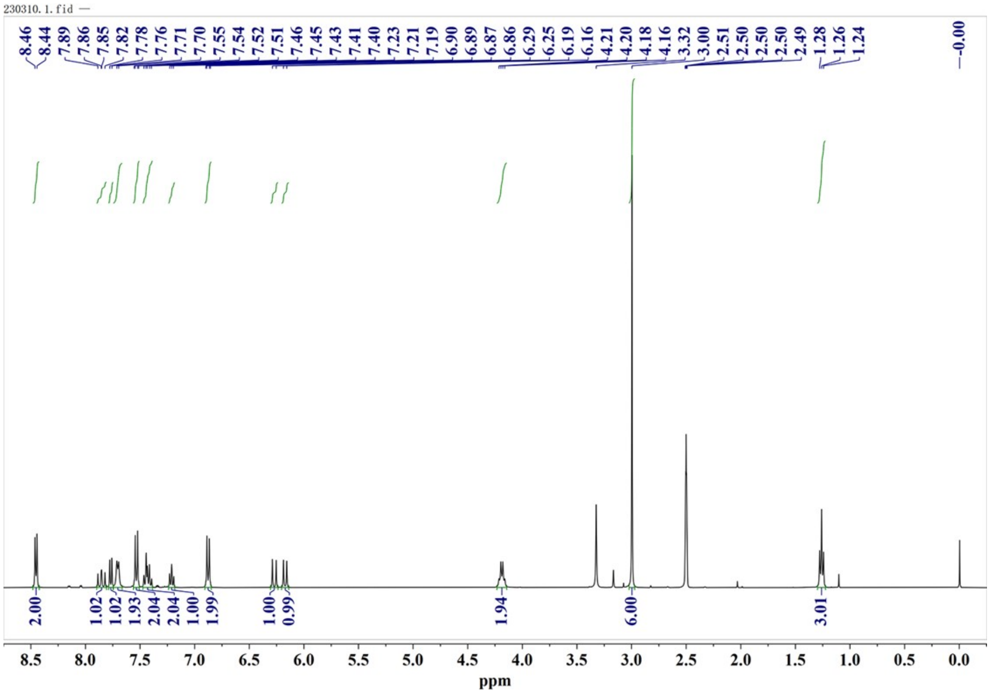

Figure S19. ^1H NMR spectrum of **BPC2** in DMSO-d_6.

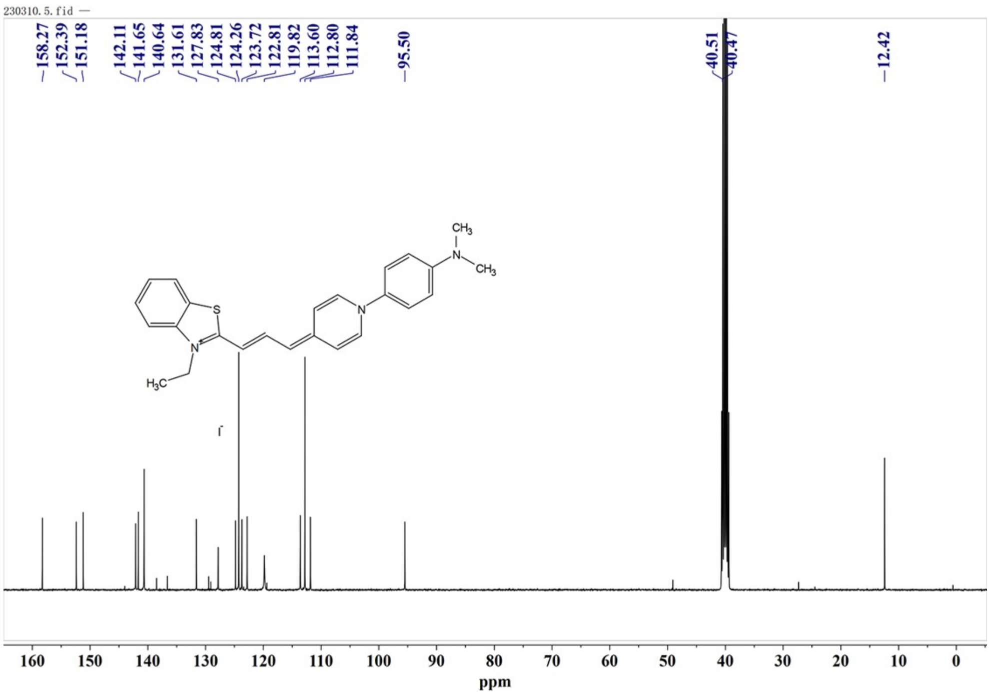

Figure S20. ^{13}C NMR spectrum of **BPC2** in DMSO-d_6.

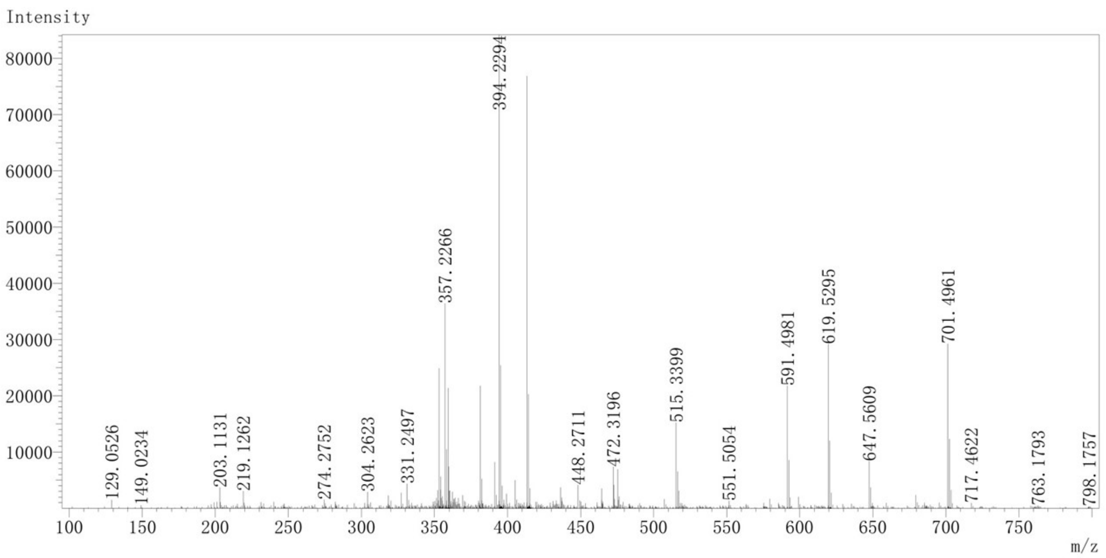

Figure S21. HR-MS spectrum of **QPC1**.

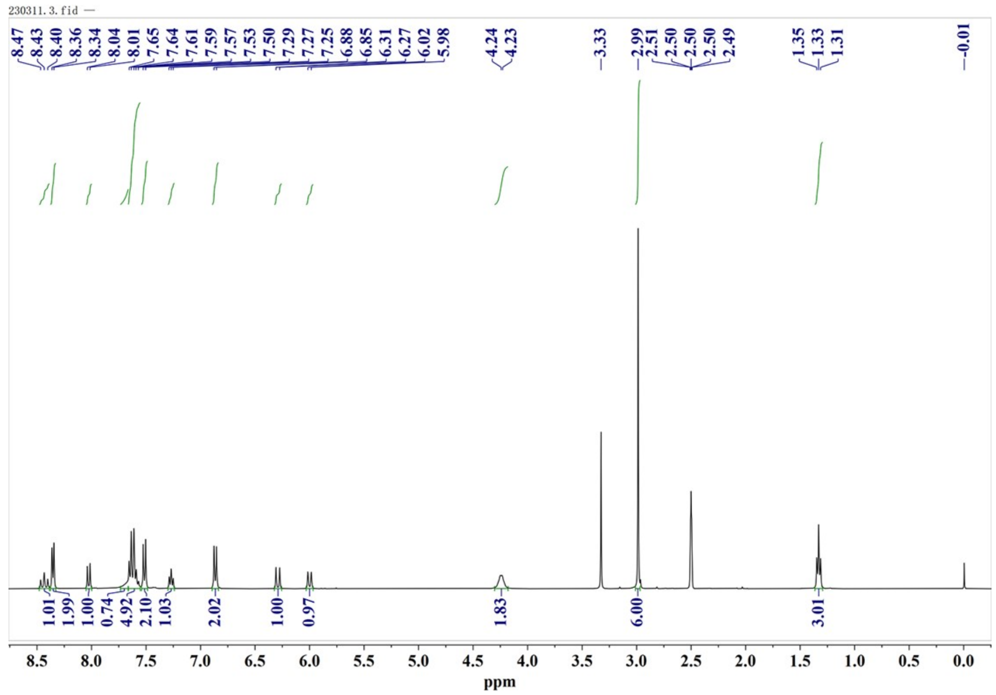

Figure S22. ^{1}H NMR spectrum of **QPC1** in DMSO-d_6.

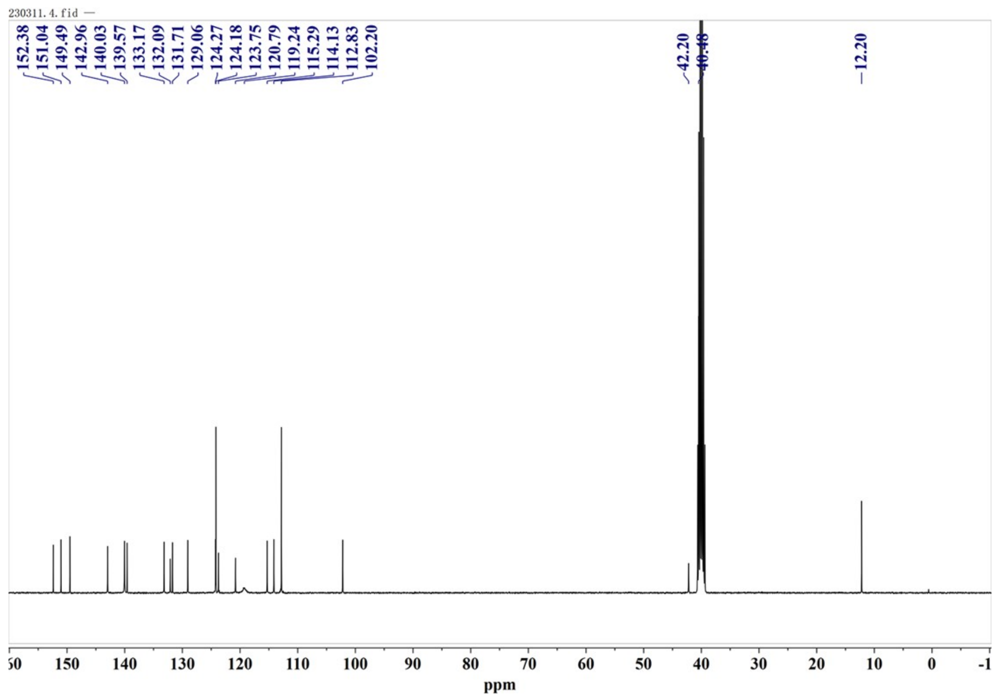

Figure S23. ^{13}C NMR spectrum of **QPC1** in DMSO-d_6.

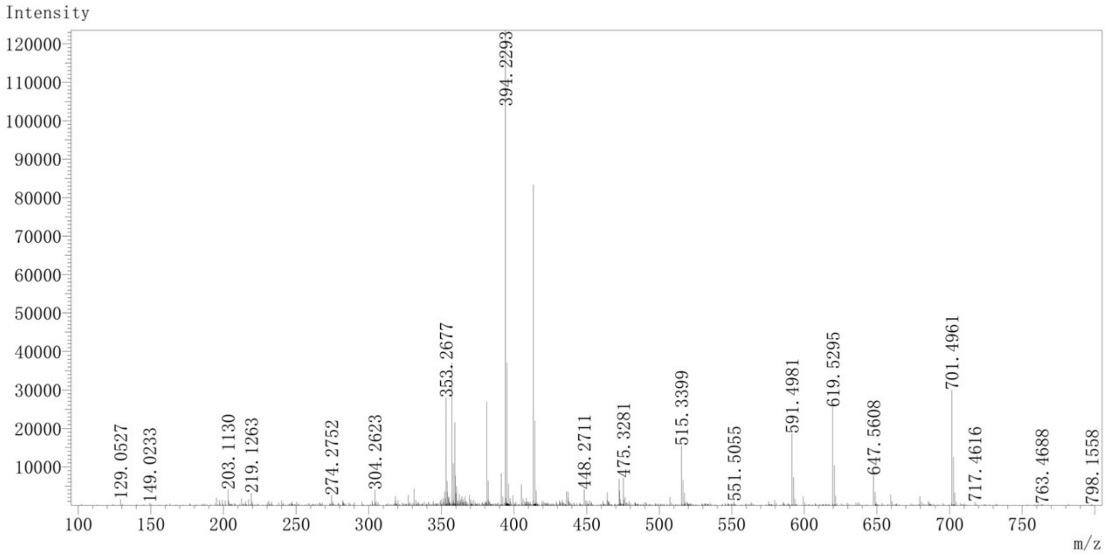

Figure S24. HR-MS spectrum of **QPC2**.

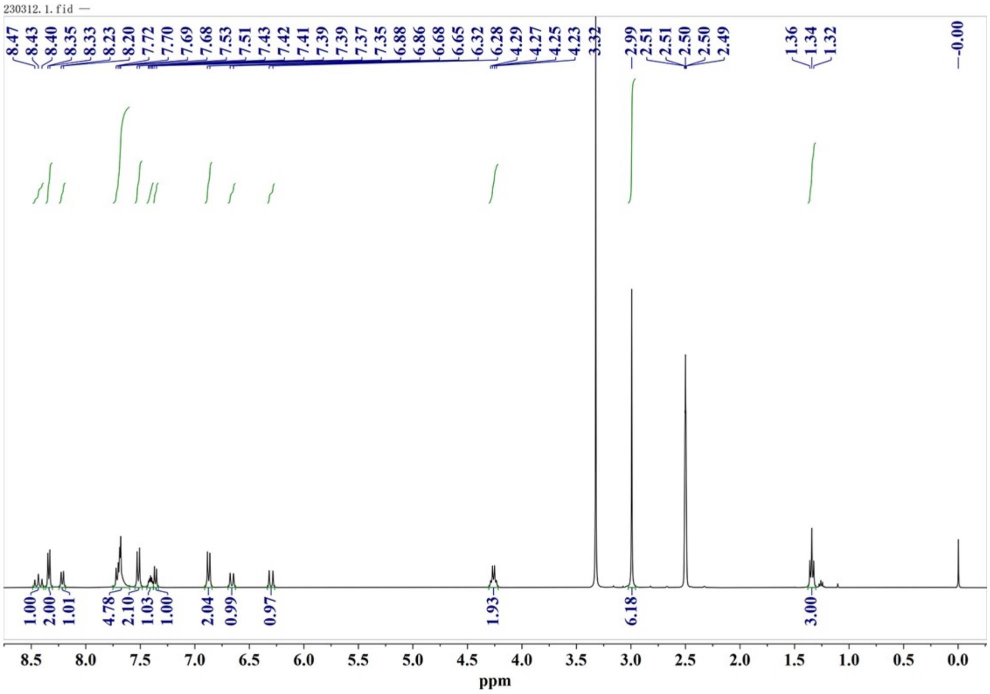

Figure S25. ^{1}H NMR spectrum of **QPC2** in DMSO-d_6.

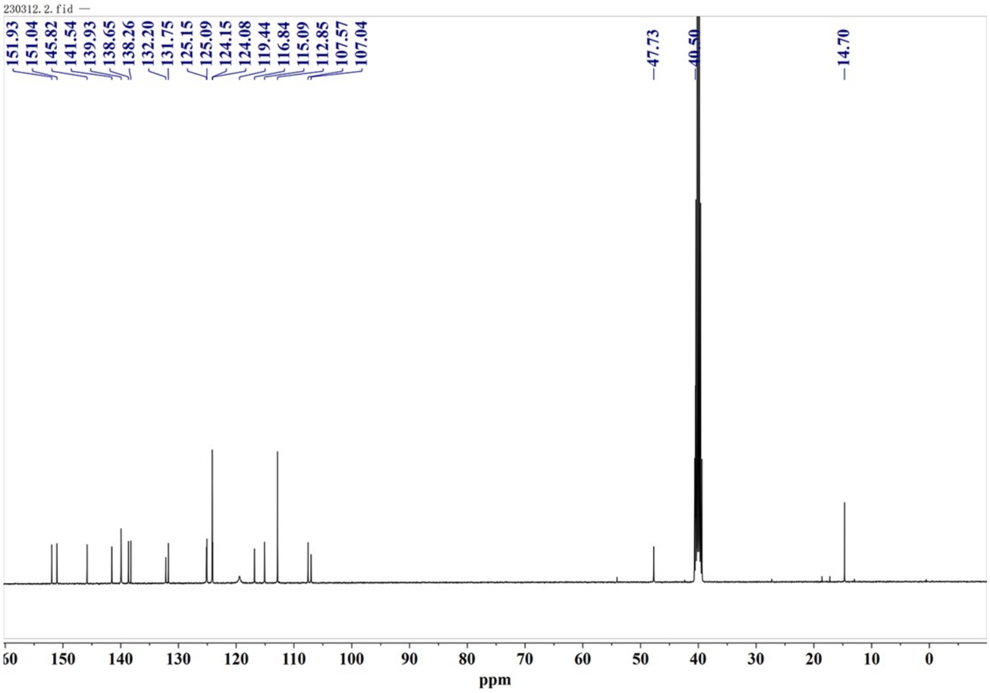

Figure S26. ^{13}C NMR spectrum of **QPC2** in DMSO-d_6.

5. Supporting reference

1. K. Uno, N. Sugimoto, Y. Sato, Nat. Commun. 2021, 12, 2650-2658.
2. Y. Li, G. V. Baryshnikov, F. Siddique, et al., Angew. Chem. Int. Ed. 2022, 61,

e202213051.

3. D. Magde, R. Wong and P. G. Seybold, Photochem. Photobiol., 2002, 75, 327-334.

4. C. Zhou, Y. Liu, Z. Sun, H. Liu, L. Xu, D. Hu and J. Hu, Dyes Pigments, 2022, 205, 110488.
